# Renewable and recyclable covalent adaptable networks based on bio-derived lipoic acid†

**DOI:** 10.1039/d1py00754h

**Published:** 2021-09-17

**Authors:** Maher A. Alraddadi, Viviane Chiaradia, Connor J. Stubbs, Joshua C. Worch, Andrew P. Dove

**Affiliations:** School of Chemistry, University of Birmingham Edgbaston B15 2TT UK j.worch@bham.ac.uk a.dove@bham.ac.uk

## Abstract

The modern materials economy is inefficient since most products are principally derived from non-renewable feedstocks and largely single-use in nature. Conventional thermoset materials are often inherently unreprocessable due to their irreversible covalent crosslinks and hence are challenging to recycle and/or reprocess. Covalent adaptable networks (CAN)s, which incorporate reversible or dynamic covalent bonding, have emerged as an efficient means to afford reprocessable crosslinked materials and increasing the feedstock sustainability of CANs is a developing aim. In this study, the biomass-derived lipoic acid, which possesses a dynamic cyclic disulfide moiety, was transformed into a series of bifunctional monomers *via* a one-step esterification or amidation reaction and reacted with a commercially available multi-valent thiol in the presence of an organobase catalyst to afford dynamically crosslinked networks. Large differences in material properties, such as storage modulus and glass transition temperature, were observed when the ratio of the lipoic acid-based monomer to thiol (from 1 : 1 to 16 : 1) and the composition of the monomer were changed to modify the network architecture. The thermomechanical properties of an optimised formulation were investigated more thoroughly to reveal a moderately strong rubber (ultimate tensile strength = 1.8 ± 0.4 MPa) possessing a large rubbery plateau (from 0 to 150 °C) which provides an adaptable material with a wide operational temperature range. Finally, the chemical recycling, or depolymerisation, of the optimised network was also demonstrated by simply solvating the material in the presence of an organobase catalyst.

## Abstract 2

Lipoic acid provides a modular and bio-sourced platform from which the create covalent adaptable network materials.

## Graphical abstract

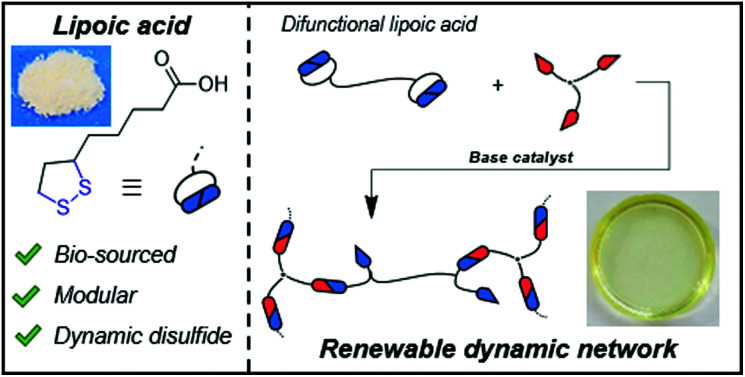

## Introduction

Developing materials that are more compatible for reprocessing and/or reformulation is central to addressing the global environmental challenges linked to the abundant use of modern plastics and thermosets. Covalent adaptable network (CAN) materials,^1–4^ which feature dynamic covalent bonding^5–7^ at crosslinking sites, seek to combine the reprocessibility advantage that is inherent to thermoplastics with the mechanical robustness of conventional thermosets. However, the implementation of sustainable feedstocks is also needed in order to transition from petroleum-derived platform chemicals that have dominated the modern materials economy, alongside research in the CAN material space.^8^ Progress on this front has relied on the derivatisation of bio-derived chemicals to enable dynamic bonding with the most common examples of bio-derived CAN materials featuring imine^9–16^ and epoxy^17–23^ chemistries. On the other hand, the use of a renewably sourced monomer that possesses inherent dynamic functionality is a more attractive feature for next-generation CAN materials since synthetic costs could be mitigated and overall sustainability improved.

Thioctic acid, or lipoic acid, is a naturally occurring carboxylic acid that contains a 5-membered cyclic disulfide which can be polymerised into a polydisulfide by ring-opening polymerisation (ROP).^24^ The ROP of various strained cyclic disulfides to afford linear polydisulfides is well established and versatile as exemplified by numerous reaction pathways – such as thermal,^25^ radical^26,27^ or anionic (*via* thiolate).^28–34^ An exemplary feature of strained cyclic disulfides is their dynamic nature that results from facile thiol-disulfide exchange^35–38^ and has been exploited to reversibly depolymerise (or chemically recycle) some of the aforementioned polydisulfides^27,29–31,33^ or to create functional supramolecular cyclic structures.^39^

Very recently, the bifunctionality and dynamicity of lipoic acid has been investigated to synthesise supramolecular crosslinked polymers by first polymerising through the cyclic disulfide (*i.e.* ROP) and then crosslinking the material *via* metal coordination/complexation of the carboxylate moiety.^40–46^ Other studies have also modified the carboxylic acid of lipoic acid with dopamine^47^ or *N*-hydroxy succinimide^48^ to produce self-healing supramolecular adhesives. Additionally, dynamic cyclic disulfides have also featured in degradable nanoparticles^49,50^ and adaptable hydrogels,^51–54^ although in the gel examples the disulfide features as a reactive polymer pendant group or chain-end and thus the systems are synthetically non-trivial and more complex. To date, the creation of a simple, non-swollen dynamic material where the crosslinking is both driven by the thiol-disulfide exchange and features the disulfides in the polymer backbone has not been reported. However, this approach should offer easy access to mechanically robust and highly tunable dynamic materials.

Herein, we created a series of structurally simple disulfide CANs by employing synthetically accessible dimerised lipoic acid monomers directly in the crosslinking step during material formulation. The synthesis of networks with a range of properties were conveniently accessed by simply mixing a multivalent thiol, a difunctional lipoic acid-based monomer and organobase in solution under ambient conditions before pouring the mixture onto a flat surface or mould to produce a stable crosslinked film after the solvent evaporated. The dynamic networks were all amorphous with glass transition temperatures well below ambient environments, affording rubbery behaviour. Finally, the networks could be efficiently degraded (or chemically recycled) by simple dilution in solvent containing catalytic organobase.

## Results and discussion

**Fig. 1:**
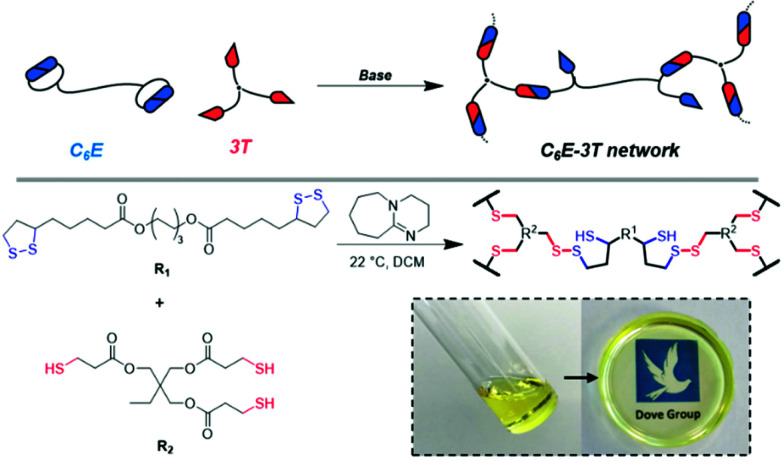
Schematic illustrating the synthesis of disulfide-based CAN (C_6_E-3T) assuming 1 : 1 disulfide to thiol. Inset shows photograph of monomer solution and subsequent crosslinking to afford homogeneous film after solvent evaporation.

We initially synthesised a dimerised ester-based monomer (C_6_E) *via* an EDC coupling of the carboxylic acid group on lipoic acid that was inspired by a recent report on similar structures for the synthesis of nanoparticles (ESI, Fig. S1 and 2†).^49^ The organobase-catalysed reaction of C_6_E with a commercial tri-armed thiol (3T) (at cyclic disulfide : thiol 1 : 1) in dichloromethane (DCM) afforded a free-standing crosslinked film (C_6_E-3T-1 : 1) after the solvent evaporated (Fig. 1).

This process is analogous to previous observations regarding the anionic ROP of lipoic acid^31^ and other disulfides^33^ that suggest the equilibrium between monomer and polymer is highly dependent upon reaction concentration. Here, the monomer is favoured under dilute conditions whereas the crosslinked material is favoured under more concentrated conditions, *i.e.* as the solvent evaporates. This process is also easily reversible for this system and networks can be chemically recycled (or depolymerised) by the addition of solvent that contains DBU (ESI, Table S1, Videos S1 and 2†). Moreover, our disulfide network was stable under ambient conditions without any further treatment. Previous studies have noted poor stability for polydisulfides unless the propagating thiolate species is protonated,^33^ quenched with an electrophile^31^ or the thiyl radical chain-end is reacted with a capping agent^41,46–48^ to form a new S–C bond. However, in our system we only employ 1 mol% base (relative to the disulfide) so not all crosslink sites could be expected to be in their ring-opened thiolate form which may explain the observed behaviour here. Fourier transform infrared spectroscopy (FTIR) experiments do not, however, indicate any S–H species in the materials (absence of absorption near 2500 cm^−1^, ESI, Fig. S9–11†).

**Fig. 2:**
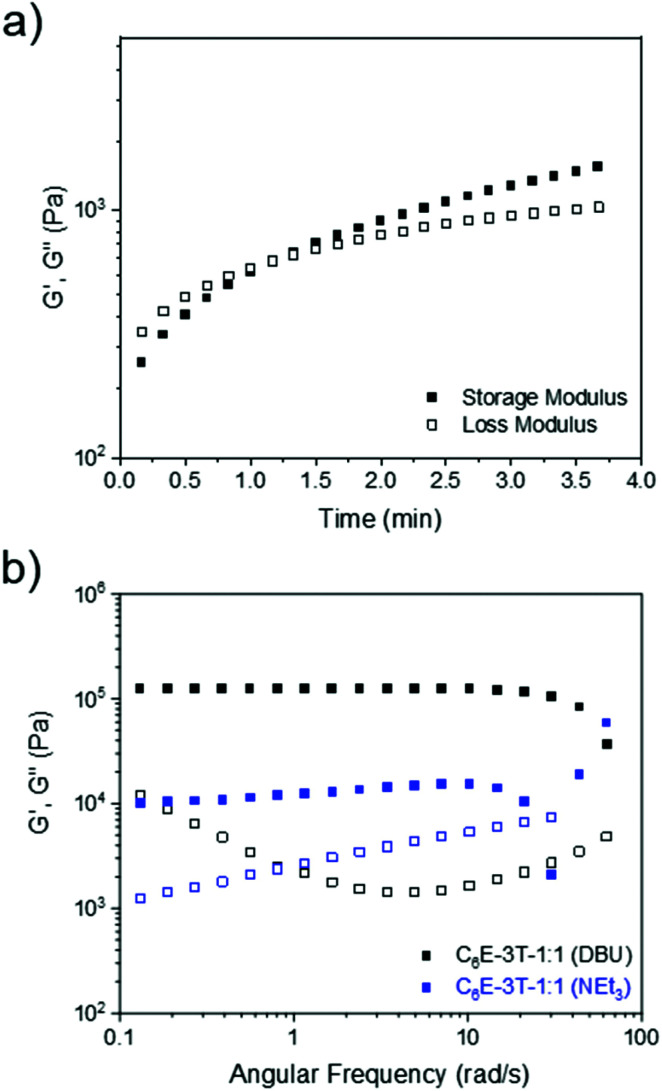
(a) Rheology time sweep (10% strain, frequency of 1 Hz) at 25 °C to characterise gelation time of C_6_E-3T-1 : 1 using 1 mol% DBU. (b) Frequency sweep analysis showing storage and loss modulus *versus* frequency for networks synthesized using DBU or NEt_3_ at 25 °C (1% strain from 0.1 to 10 Hz, or 0.06 to 62 rad s^−1^).

Although the gelation times were rapid (under 60 s) when employing NEt_3_ or DBU (ranging from 1–5 mol% relative to the thiol) the films produced from NEt_3_ were tacky in nature, even after the solvent had fully evaporated, and thus DBU (1 mol% loading) was selected as the optimal catalyst (Fig. 2a). This observation was corroborated by the frequency sweep rheological analysis of two films synthesised using either DBU or NEt_3_. Regarding the material obtained from NEt_3_, some storage modulus-frequency dependence was apparent, especially at higher rates, which suggests a less solid-like structure (Fig. 2b). Dimethyl carbonate, which is a greener and more polar solvent,^55,56^ was also screened but the gelation occurred almost instantly. Nevertheless, a wide range of solvents were found to facilitate the reaction and lowering the amount of base catalyst could allow for better film formation when using polar (and/or greener) solvents. When using chloroform or DCM, the films had similar physical appearance and gelation times so the latter solvent was preferred since it has a lower boiling point.

**Fig. 3:**
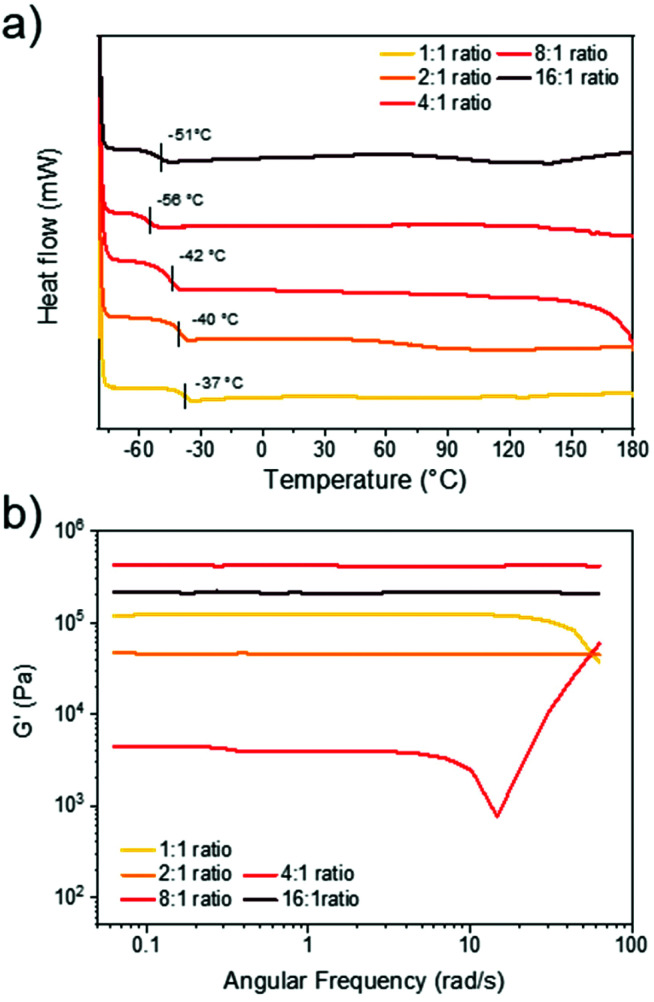
Thermal and rheological properties of C_6_E-3T networks. (a) DSC thermograms of the 2nd heating cycle from −80 to 180 °C, 10 °C min^−1^. (b) Frequency sweep analysis of films showing storage modulus *versus* frequency at 25 °C (1% strain from 0.1 to 10 Hz, or 0.06 to 62 rad s^−1^).

After optimising the reaction conditions for the synthesis of the 1 : 1 disulfide network, we set out to expand the material library. By altering the relative ratio of disulfide to thiol, it was possible to change the crosslinking density which is an important design consideration in dynamic material synthesis.^57,58^ By increasing the stoichiometry of disulfide to thiol, oligomeric units could theoretically form (assuming idealised reactivity where each thiol initiates a disulfide monomer to form a new thiolate species). This could be expected to lead to longer chains between crosslinks and/or increased branching in the network, and thus tune the bulk properties of the materials. Using the optimised reaction conditions (disulfide 1 M in DCM, 1 mol% DBU), all synthesised networks exhibited rapid gelation times (under 60 s) and formed homogenous films. Expanding upon the initial system (C_6_E-3T-1 : 1), we produced networks with higher disulfide contents (C_6_E-3T-4 : 1, C_6_E-3T-8 : 1) and compared the thermal properties of these films to C_6_E-3T-1 : 1 using differential scanning calorimetry (DSC) (Fig. 3a). All materials were amorphous and in the rubbery regime under ambient conditions as evidenced by their low glass transition temperatures (*T*_g_ = −37 °C to −56 °C). The most densely crosslinked material (1 : 1 disulfide to thiol) exhibited the highest *T*_g_. In general, the *T*_g_ decreased as the ratio of disulfide to thiol increased but C_6_E-3T-16 : 1 had a slightly higher value than C_6_E-3T-8 : 1. The DSC data suggests that the overall network topology was significantly modulated by adjusting monomer stoichiometry, although the precise network structure cannot be elucidated. Branching architectures may contribute to lowering the glass transition temperature in addition to overall crosslinking density.

The rheological properties of the various C_6_E-3T networks were also investigated. Using a frequency sweep analysis at 25 °C, the 16 : 1, 8 : 1, and 2 : 1 ratios displayed unchanging elastic (solid-like) behaviour (Fig. 3b). However, the 1 : 1 and 4 : 1 ratios exhibited some frequency dependence, suggesting more fluid-like behaviour (Fig. 3b). Strain sweep experiments of the 4 : 1 ratio also suggest poor structural integrity of the film since the storage modulus is highly strain-dependent (ESI, Fig. S16†). The storage modulus of the samples differed by an order of magnitude according to their stoichiometry and the 4 : 1 ratio (C_6_E-3T-4 : 1 = 4.4 kPa) was lower than the initial 1 : 1 ratio (C_6_E-3T-1 : 1 = 120 kPa), but increased significantly for the 8 : 1 ratio material (C_6_E-3T-8 : 1 = 431 kPa). Again, these results suggest that for C_6_E-3T-4 : 1 system there is relatively inefficient crosslinking, *i.e.* poor network topology. Interestingly, the modulus for C_6_E-3T-2 : 1 (47 kPa) was also lower than C_6_E-3T-1 : 1, with a similar trend observed for C_6_E-3T-16 : 1 (213 kPa) compared to C_6_E-3T-8 : 1 (Fig. 3b). Together these data, along with thermal characteristics, indicate that the material properties can be significantly changed by adjusting relative stoichiometry of the disulfide to thiol. We are still investigating the superior properties that were observed in the 8 : 1 ratio material. There could be a ‘Goldilocks zone’ for this CAN system when the disulfide to thiol ratio is near 8 : 1, *i.e.* the 1 : 2 and 1 : 4 materials were relatively poor and the 16 : 1 ratio network was slightly less robust in comparison. A possible explanation points toward an advantageous effect from branching, up to a certain extent, likely somewhere between the 8 : 1 and 16 : 1 formulations.

**Fig. 4:**
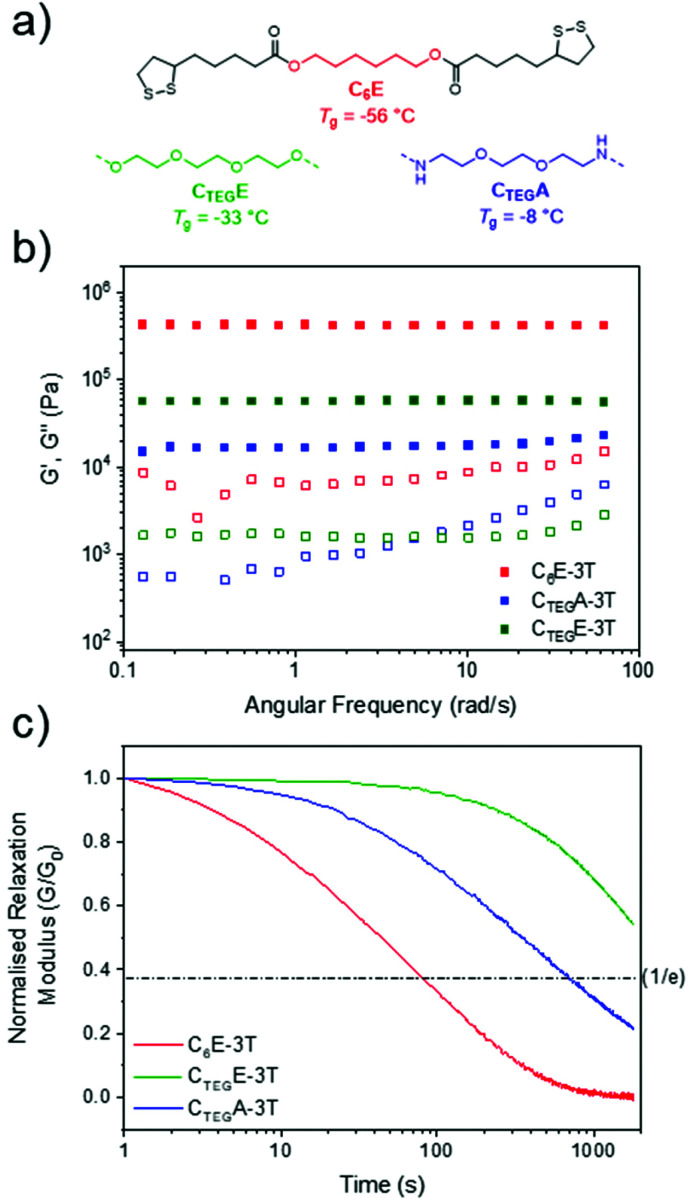
(a) Structures of monomers to make networks at 8 : 1 ratio. (b) Rheology frequency sweep of networks at 25 °C (1% strain from 0.1 to 10 Hz, or 0.06 to 62 rad s^−1^). (c) Stress relaxation experiments at 2% strain, 100 °C.

Next, we investigated other dimeric monomers which were also synthesised in one step from lipoic acid using an EDC coupling strategy. An analogous amide to C_6_E was synthesised from 1,6-hexanediamine (C_6_A) in addition to derivatives containing more flexible ether units formed from triethylene glycol (C_TEG_E) and 1,8-diamino-3,6-dioxaoctane (C_TEG_A) (Fig. 4a and ESI, Fig. S3–8†). However, we were unable to produce films from C_6_A due to its poor solubility. These compositionally distinct networks were also synthesised at various ratios (1 : 1, 4 : 1, 8 : 1) to directly compare with materials from the alkyl monomer (C_6_E) series. For the glycol ester-based materials (C_TEG_E-3T), the 1 : 1 ratio network had a similar glass transition temperature to the C_6_E-3T analogue, but there was little variation according to monomer stoichiometry (Δ*T*_g_ = 6 °C) and C_TEG_E-3T-8 : 1 had the highest *T*_g_ of −33 °C within the series (ESI, Fig. S12†). The amide-based materials (C_TEG_A-3T) provided some H-bonding character to the network which likely contributes to higher glass transition temperatures (*T*_g_ = −28 °C to 4 °C) compared to the ester variants, with the 1 : 1 ratio material also presenting with the highest value (ESI, Fig. S12†). The H-bonding is supported by a broad absorption near 3290 cm^−1^ with a small shoulder signal (3070 cm^−1^) in the FTIR spectra for the series (ESI, Fig. S11†). As such, it is difficult to define any general trends in the thermal properties among networks with different compositions. However, all of the CANs displayed high thermal stabilities with decomposition temperatures (*T*_d,5%_) ranging from 234–277 °C (ESI, Fig. S13–15†). The disulfide monomer content was also positively correlated to the decomposition temperatures (Table S2†).

Comparing the three different monomer systems at the same ratio (8 : 1) offers striking differences in their rheological behaviour (Fig. 4). When investigating the analogous glycol monomer (C_TEG_E-3T-8 : 1), the material was considerably less stiff as compared to the initial system (C_6_E-3T-8 : 1) as evidenced by a lower storage modulus (56 kPa) (Fig. 4b). However, the amide version (C_TEG_A-3T-8 : 1) possessed the lowest modulus in the series, at only 15 kPa, which is a contrast to what might be expected on account of the presence of H-bonding interactions. It is possible that the H-bonding may be disturbed by the complexity of the material topology. Interestingly, the C_6_E and C_TEG_A networks both exhibited rapid stress relaxation at 100 °C which alludes to their reprocessibility (Fig. 4c). Full stress relaxation is observed for both CANs as a result of base-assisted disulfide bond exchange and/or reversible formation of the cyclic disulfide. Moreover, C_6_E-3T-8 : 1 shows a full relaxation time (*i.e.* where the material may theoretically flow for reprocessing) of only 80 s at 100 °C. The full relaxation time is determined to be the time corresponding to (1/*e*) or ∼37% of the initial stress value on normalised relaxation modulus (*G*/*G*_0_) based on the Maxwell model.^59^ At the same temperature, the C_TEG_A network relaxed approximately an order of magnitude slower (full relaxation time ≈ 700 s), which could be due to additional H-bonding interactions from the amide moiety. The higher *T*_g's_ observed in the amide networks also supports this observation (ESI, Fig. S12†). However, the C_TEG_E material had the slowest relaxation kinetics of the series and full relaxation was not observed within the experimental time frame (Fig. 4c). When compared to the amide structure, this result is surprising considering the relatively low *T*_g_ and lack of obvious additional H-bonding interactions. It is possible that the rate of the disulfide exchange or formation of the cyclic disulfide, and thus the overall dynamicity of the system, is influenced by the nature of the linking group (*i.e.* alkyl *vs*. ether and/or ester *vs*. amide). Finally, the trend in rheological properties within each respective class of materials also differed according to stoichiometry. For example, in the C_TEG_E system the 4 : 1 ratio had a higher modulus than the 1 : 1 ratio and the amides (C_TEG_A-3T) generally followed the same trend, although both were disimilar to the C_6_E networks (ESI, Fig. S20–24†).

**Fig. 5:**
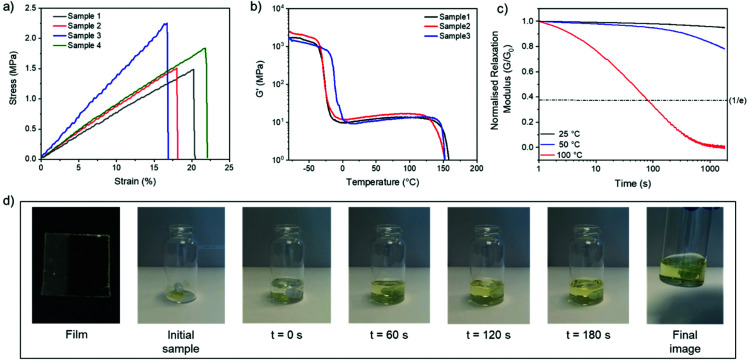
Thermomechanical and physical data for C6E-3T-8 : 1. (a) Uniaxial tensile testing at 22 °C, 10 mm min^−1^ strain rate. (b) DMA temperature sweep from −80 to 180 °C, 5 °C min^−1^. (C) Stress relaxation experiments at 2% strain (25 °C, 50 °C, and 100 °C). (d) Chemical recycling of as-synthesised film cut into small pieces (200 mg film in 5 mL DCM solution, 0.01 M DBU).

After screening various disulfide CANs, we selected C_6_E-3T-8 : 1 for further investigation based on its optimal thermal (lowest *T*_g_) and rheological (highest storage modulus and excellent relaxation behaviour) properties. The mechanical properties were initially investigated using uniaxial tensile testing (Fig. 5a). The synthesised material can best be described as a rubber (Young's modulus = 9.1 ± 1.9 MPa) with moderate ultimate tensile strength (UTS = 1.8 ± 0.4 MPa) and a modest elongation at break (19 ± 2%) (Table S3†). The material properties *versus* temperature were then studied using dynamic mechanical analysis (DMA), revealing a large rubbery plateau before bulk flow of the material was observed near 150 °C (Fig. 5b and ESI, Fig. S28†). Additional stress relaxation experiments conducted at various temperatures revealed that the material was relatively stable at low to moderate temperatures (25 °C or 50 °C), but exhibited a sharp decrease in viscosity at 100 °C (Fig. 5c, see ESI† for other networks at various temperatures).

**Fig. 6:**
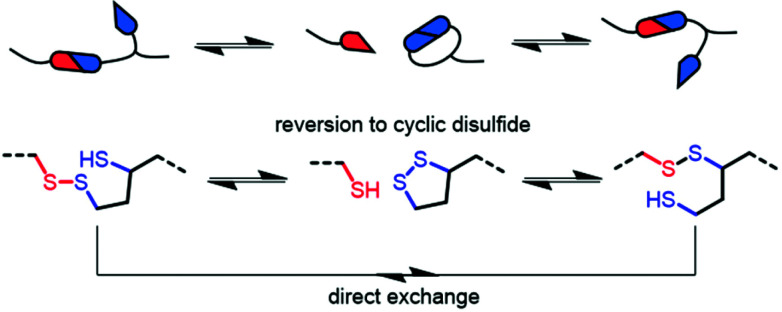
Possible base-catalysed disulfide reaction pathways in networks.

The dynamic behaviour was further probed by thermally reprocessing the material at 180 °C. Even though the material flowed to form a new homogeneous film after cooling, it was tacky in nature (ESI, Fig. S27†) indicating that the network did not completely reform. At elevated temperatures, each linear disulfide unit at a crosslink point can likely react by either direct exchange or reversion to the cyclic disulfide (Fig. 6). Then, reformation of an idealised linear disulfide network could be realised *via* a nucleophilic ring-opening route due to the presence of residual DBU within the system. However, it is possible that the DBU was thermally degraded at 180 °C and thus the bulk material had inadequate catalyst to facilitate the subsequent reformation of the crosslinked structure. A photo-mediated radical exchange mechanism is unlikely since a high intensity UV-light source (≥60 mW cm^−2^) was required to enable dynamic exchange in another lipoic acid-based polymer.^27^ Furthermore, our materials were observed to be stable under ambient light over a period of at least several weeks. Nevertheless, efficient chemical recycling of the network was demonstrated by adding a solution of DCM containing DBU to the film, thus showcasing its adaptive nature (Fig. 5d, ESI, Table S1, Videos S1 and 2†). The network briefly swelled in the solvent, but was fully dissolved within 3 min after adding 0.01 M DBU solution.

## Conclusions

A library of dynamic networks based on disulfide chemistry were prepared from synthetically accessible bifunctional lipoic acid-derived monomers. By varying network composition *via* monomer exchange and crosslinking ratios, we were able to obtain materials with divergent thermal and rheological properties. However, when adjusting the stoichiometry to alter the crosslinking density, the trends in material performance differed significantly depending on the starting monomer which precluded any universal structure–property relationships for this study. Future experiments are underway to more thoroughly investigate the network topologies which appear to be complex (*i.e.* non-idealised) and could be obfuscated by branching regions. Nevertheless, all synthesised materials can be characterised as soft and rubbery with good structural integrity at ambient temperatures. C_6_E_8_-3T was found to exhibit optimal material properties and more in-depth thermomechanical analysis using DMA and tensile testing revealed a moderately strong rubber with a rubbery plateau extending out to around 150 °C. Although thermal reprocessing of the film revealed a tacky material, this is likely a result of inefficient reformation of the network due to thermal degradation of the amine base. Future experiments are underway to enhance the thermal reprocessability of these dynamic networks. Finally, base-promoted chemical recycling of C_6_E-3T samples was also demonstrated which highlights the versatility of the disulfide CANs as renewable dynamic materials.

## Author contributions

J. C. W. and A. P. D. conceived the material synthesis and designed the project idea. J. C. W. synthesised monomers. C. J. S. performed TGA and FTIR analysis. M. A. and V. C. synthesised the networks and performed rheology, DSC, and tensile experiments. M. A. and V. C. contributed equally. The paper was written through contributions of J. C. W. and A. P. D. All authors have given approval to the final version of the paper.

## Conflicts of interest

There are no conflicts to declare.

## Supplementary Material

PY-012-D1PY00754H-s001

PY-012-D1PY00754H-s002

PY-012-D1PY00754H-s003
