# Supplementary material for: Renewable and recyclable covalent adaptable networks based on bio-derived lipoic acid
Source: Polym Chem. 2021 Sep 17;12(40):5796–802. doi: 10.1039/d1py00754h (PMC8524469; doi:10.1039/d1py00754h)
Supplement: PY-012-D1PY00754H-s001 [file PY-012-D1PY00754H-s001.pdf]

Electronic Supplementary Material (ESI) for Polymer Chemistry.
This journal is © The Royal Society of Chemistry 2021

Renewable covalent adaptable networks based on bio-derived lipoic acid

Maher A. Alraddadi†[a], Viviane Chiaradia†[a], Connor J. Stubbs[a], Joshua C. Worch*[a] and Andrew P. Dove*[a]

[a] *School of Chemistry, University of Birmingham, Edgbaston B15 2TT, United Kingdom*

Corresponding Authors: adove@bham.ac.uk; jworch@bham.ac.uk

Table of Contents

Materials and methods........S1-S3

Experimental procedures........S3-S6

NMR spectra of monomers........S6-S10

FTIR spectra of networks........S10-S11

DSC thermograms of polymers........S12

TGA thermograms of polymers........S13-S14

Rheology data to assess material properties of networks........S15-S19

Thermal Reprocessing of C_6E-3T-8:1 network........S20

Chemical recycling of C_6E-3T networks........S20

DMA data of C_6E-3T-8:1 network........S21

Summary of physical, thermal and mechanical properties for all networks........S22

Summary of DMA and tensile data for C_6E-3T-8:1 network........S22

References........S22

General Materials and Methods. All compounds, unless otherwise indicated, were purchased from commercial sources and used as received. All solvents and chemicals used for recrystallisation were used as received. All films were prepared under ambient conditions and left overnight *ca.* 16 h in order to remove residual solvent before testing.

NMR Spectroscopic Analysis. All NMR spectroscopy experiments were performed at 298 K on a Bruker DPX-400 NMR instrument equipped operating at 400 MHz for ^{1}H (100.57 MHz for ^{13}C). ^{1}H NMR spectra are referenced to residual protio solvent (δ = 7.26 for $CDCl_3$) and ^{13}C NMR spectra are referenced to the

solvent signal (δ = 77.16 for $CDCl_3$). The resonance multiplicities are described as s (singlet), d (doublet), t (triplet), q (quartet) or m (multiplet).

Mass Spectrometry. High Resolution Electrospray Ionization Mass Spectrometry was performed in the School of Chemistry at University of Birmingham on a Waters Xevo G2-XS QTof Quadrupole Time-of-Flight mass spectrometer.

Differential Scanning Calorimetry (DSC). The thermal characteristics of the polymers were determined using differential scanning calorimetry (STARe system DSC3, Mettler Toledo) from −80 to 180 °C at a heating rate of 10 °C min^{-1} for two heating/cooling cycles unless otherwise specified. The glass transition temperature (T_g) was determined from the inflection point in the second heating cycle of DSC.

Thermogravimetric Analysis (TGA). TGA thermograms were obtained using a TGA/DSC 1 - Thermogravimetric Analyzer (Mettler Toledo). Thermograms were recorded under an N_2 atmosphere at a heating rate of 10 °C min^{-1}, from 10 − 600 °C, with an average sample weight of *ca*. 10 mg. Aluminium pans were used for all samples. Decomposition temperatures were reported as the 5% weight loss temperature ($T_{d\ 5\%}$).

Fourier-transform infrared (FTIR) spectroscopy. FTIR spectra were collected out using an Agilent Technologies Cary 630 FTIR spectrometer. 16 Scans from 600 to 4000 cm^{-1} were taken at a resolution of 4 cm^{-1}, and the spectra were corrected for background transmittance.

Rheology. Rheological measurements were performed on an Anton Paar MCR 302 using Anton Paar PP8 parallel-plate, a diameter of 8 mm. Temperature was controlled with a P-PTD 200/AIR Peltier and a P-PTD 200 hood. Gelation time was monitored at 10% strain, frequency of 1 Hz and 0 N of normal force. Frequency sweeps were performed at 1% strain from 0.01 to 10 Hz (0.06 to 62 rad/s). Amplitude sweeps were performed at 1 Hz from 0.01 to 500%. Stress relaxation tests were performed at 2% strain at 25 °C, 50 °C, and 100 °C. Sample thickness was approximately 1 mm.

Dynamic Mechanical Analysis (DMA). Dynamic mechanical thermal analysis (DMTA) data were obtained using a Mettler Toledo DMA 1 star system and analyzed using the software package STARe V13.00a (build 6917). Thermal sweeps were conducted using films (L x W x thickness = 15.08 mm x 6.16 mm x 0.30 mm) cooled to −80°C and held isothermally for *ca*. 5 minutes. Storage and loss moduli, as well as the loss factor (ratio of *E"* and *E'*, tan δ) were probed as the temperature was swept from −80 to 180 °C, 5 °C min^{-1}, 1 Hz. Thermomechanical behavior was determined from three samples in this way.

Uniaxial Tensile Testing. Dumbbell-shaped samples were cut directly from the synthesized films using a custom ASTM Die D-638 Type V. Tensile tests at different stretching speed were carried out using a Testometric M350-5CT universal mechanical testing instrument fitted with a load cell of 5 kN at room temperature (22 ± 1 °C). The gauge length was set as 7.1 mm and the crosshead speed was set 10 mm min^{-1}. The dimensions of the neck of the specimens were 7.1 mm in length, 1.6 mm in width and 0.2 mm in thickness. The reported results are average values from at least three individual measurements (n ≥ 3).

Chemical recycling studies. The network film was manually cut into small pieces, placed into 20 mL scintillation vial equipped with a stirrer bar and then diluted with DCM containing DBU. For reactions using 0.01 M DBU solution, 200 mg of sample was diluted with 5 mL of solution. For reactions using 0.05 M DBU solution, 150 mg of sample was diluted with 3.75 mL of solution. The reaction mixture was then stirred at ambient temperature (22 ± 1 °C) and the degradation of the network was monitored using video recording until the solution was homogeneous.

Experimental Procedures

C_6E Synthesis

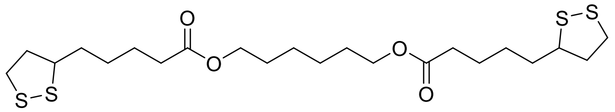

Lipoic acid (10 g, 2 equiv., 48.4 mmol), 1,6-hexanediol (2.86 g, 1 equiv., 24.2 mmol), DMAP (5.91 g, 1 equiv., 48.4 mmol) were placed in an oven-dried 250 mL 2-neck round-bottom flask and back-filled with N_2. DCM (100 mL) was added to the flask and the mixture was stirred until all reagents were dissolved (*ca.* 10 min). Note: the DCM did not have to be rigorously dried and reagent grade solvent was adequate for the reaction. The reaction mixture was then cooled to 0 °C in an ice-water bath and EDC·HCl (9.28 g, 1 equiv., 48.4 mmol) was added portion-wise over 5 min. After the addition was complete, the reaction was stirred for 15 min at 0 °C, then removed from the ice-bath. The flask was wrapped in aluminum foil to protect from ambient light and stirred overnight at ambient temperature (*ca.* 16 h at 22 °C). The reaction mixture was transferred to a 250 mL round-bottom flask and concentrated *in vacuo*. The crude mixture was purified directly using silica gel column chromatography, eluting with $CHCl_3$/MeOH 40/1 (R_f ≈ 0.7 $CHCl_3$/MeOH, 40/1) to afford a yellow oil after concentration *in vacuo* (yield = 9.72 g, 81%). ^1H NMR (400 MHz, Chloroform-*d*) δ 4.04 (t, J = 6.7 Hz, 4H), 3.55 (m, 2H), 3.27 – 2.98 (m, 4H), 2.56 – 2.35 (m, 2H), 2.29 (t, J = 7.4, 2H), 1.89 (m, 2H), 1.79 – 1.53 (m, 6H), 1.55 – 1.13 (m, 4H). ^{13}C NMR (101 MHz, Chloroform-*d*) δ 173.61, 77.48, 77.16, 76.84, 64.34, 56.42, 40.30, 38.57, 34.68, 34.17, 28.84, 28.61, 25.69, 24.78. HRMS (ESI-TOF) (*m/z*): [M + H] calculated for $C_{22}H_{38}O_4S_4$ H 495.1731; found 495.1729. Note: sometimes undesirable crosslinking was observed upon concentration of the purified product, but this can be significantly mitigated by adding butylated hydroxytoluene (BHT) (~ 50 mg) to the solution before concentrating.

$C_{TEG}E$ Synthesis

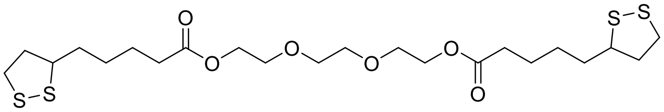

Lipoic acid (10 g, 2 equiv., 48.4 mmol), triethylene glycol (3.62 g, 1 equiv., 24.2 mmol), DMAP (5.91 g, 1 equiv., 48.4 mmol) were placed in an oven-dried 250 mL 2-neck round-bottom flask and back-filled with N_2. DCM (100 mL) was added to the flask and the mixture was stirred until all reagents were dissolved (*ca.* 10 mins). Note: the DCM did not have to be rigorously dried and reagent grade solvent was adequate for the reaction. The

reaction mixture was then cooled to 0 °C in an ice-water bath and EDC·HCl (9.28 g, 1 equiv., 48.4 mmol) was added portion-wise over 5 min. After the addition was complete, the reaction was stirred for 15 min at 0 °C, then removed from the ice-bath. The flask was wrapped in aluminum foil to protect from ambient light and stirred overnight at ambient temperature (*ca.* 16 h at 22 °C). The reaction mixture was transferred to a 250 mL round-bottom flask and concentrated *in vacuo*. The crude mixture was purified directly using silica gel column chromatography, eluting with $CHCl_3$/MeOH 20/1 ($R_f \approx 0.6$ $CHCl_3$/MeOH, 20/1) to afford a yellow oil after concentration *in vacuo* (yield = 13.05 g, quantitative). ^{1}H NMR (400 MHz, Chloroform-*d*) δ 4.26 – 4.14 (m, 4H), 3.70 – 3.65 (m, 4H), 3.63 (s, 4H), 3.54 (m, 2H), 3.23 – 3.02 (m, 4H), 2.43 (m, 2H), 2.32 (t, J = 7.4 Hz, 4H), 1.88 (m, 2H), 1.75 – 1.56 (m, 8H), 1.54 – 1.34 (m, 4H). ^{13}C NMR (101 MHz, Chloroform-*d*) δ 173.46, 77.48, 77.36, 77.16, 76.84, 70.59, 69.26, 63.45, 56.39, 40.27, 38.54, 34.64, 33.98, 28.77, 24.66. HRMS (ESI-TOF) (*m/z*): [M + H] calculated for $C_{22}H_{38}O_6S_4$ H 527.1630; found 527.1641. Note: sometimes undesirable crosslinking was observed upon concentration of the purified product, but this can be significantly mitigated by adding butylated hydroxytoluene (BHT) (~ 50 mg) to the solution before concentrating.

C_6A Synthesis

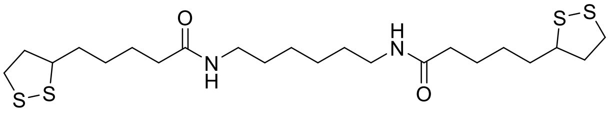

Lipoic acid (10 g, 2 equiv., 48.4 mmol), 1,6-hexanediamine (2.81 g, 1 equiv., 24.2 mmol), DMAP (5.91 g, 1 equiv., 48.4 mmol) were placed in an oven-dried 250 mL 2-neck round-bottom flask and back-filled with N_2. DCM (100 mL) was added to the flask and the mixture was stirred for *ca.* 10 min. Note: the DCM did not have to be rigorously dried and reagent grade solvent was adequate for the reaction. The reaction mixture was then cooled to 0 °C in an ice-water bath and EDC·HCl (9.28 g, 1 equiv., 48.4 mmol) was added portion-wise over 5 min. During the addition, white precipitate formed and after the addition was complete, the reaction was stirred at 0 °C, slowly warming in the bath overnight (c.a. 16 h). The reaction mixture was transferred to a 1 L separatory funnel and diluted with ~ 500 mL $CHCl_3$ (since solubility in DCM is poorer) to adequately dissolve the amide product. The organic layer was washed with 1 M HCl (3 x 200 mL), water (1 x 200 mL), saturated $NaHCO_3$ solution (3 x 200 mL), water (1 x 200 mL), and brine (1 x 200 mL). The organic layer was dried using $MgSO_4$ and concentrated *in vacuo* to reveal the title compound as a white solid (yield = 10.62 g, 89%). No further purification was necessary. ^{1}H NMR (400 MHz, Chloroform-*d*) δ 5.68 (s, 2H), 3.63 – 3.52 (m, 2H), 3.25 (q, J = 6.6 Hz, 4H), 3.22 – 3.06 (m, 4H), 2.46 (m, 2H), 2.26 – 2.16 (m, 4H), 1.91 (m, 2H), 1.76 – 1.61 (m, 8H), 1.54 – 1.40 (m, 8H), 1.35 (m, 4H). ^{13}C NMR (101 MHz, Chloroform-*d*) δ 172.91, 77.48, 77.16, 76.84, 56.63, 40.40, 39.05, 38.61, 36.67, 34.77, 29.61, 29.05, 26.05, 25.61. HRMS (ESI-TOF) (*m/z*): [M + H] calculated for $C_{22}H_{40}N_2O_2S_4$ H 493.2051; found 493.2061.

C_{TEG}A Synthesis

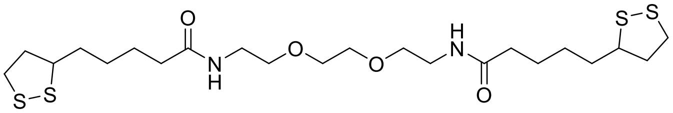

Lipoic acid (10 g, 2 equiv., 48.4 mmol), 1,8-diamino-3,6-dioxaoctane (3.58 g, 1 equiv., 24.2 mmol), DMAP (5.91 g, 1 equiv., 48.4 mmol)

were placed in an oven-dried 250 mL 2-neck round-bottom flask and back-filled with N_2. DCM (100 mL) was added to the flask and the mixture was stirred for *ca.* 10 min. Note: the DCM did not have to be rigorously dried and reagent grade solvent was adequate for the reaction. The reaction mixture was then cooled to 0 °C in an ice-water bath and EDC·HCl (9.28 g, 1 equiv., 48.4 mmol) was added portion-wise over 5 min. During the addition, white precipitate formed and after the addition was complete, the reaction was stirred at 0 °C, slowly warming in the bath overnight (c.a. 16 h). The reaction mixture was transferred to a 1 L separatory funnel and diluted with ~ 500 mL $CHCl_3$ (since solubility in DCM is poorer) to adequately dissolve the amide product. The organic layer was washed with 1 M HCl (3 x 200 mL), water (1 x 200 mL), saturated $NaHCO_3$ solution (3 x 200 mL), water (1 x 200 mL), and brine (1 x 200 mL). The organic layer was dried using $MgSO_4$ and concentrated *in vacuo* to reveal the title compound as a white solid (yield = 10.2 g, 80%). No further purification was necessary. ^{1}H NMR (400 MHz, Chloroform-*d*) δ 5.98 (s, 2H), 3.62 (s, 4H), 3.59 – 3.52 (m, 6H overlap), 3.46 (q, J = 5.2 Hz, 4H), 3.25 – 3.04 (m, 4H), 2.52 – 2.41 (m, 2H), 2.20 (t, J = 7.5 Hz, 4H), 1.97 – 1.86 (m, 2H), 1.79 – 1.59 (m, 8H), 1.59 – 1.35 (m, 4H). ^{13}C NMR (101 MHz, Chloroform-*d*) δ 172.94, 77.48, 77.16, 76.84, 70.33, 70.03, 56.54, 40.35, 39.24, 38.57, 36.48, 34.73, 28.99, 25.46. HRMS (ESI-TOF) (*m/z*): [M + H] calculated for $C_{22}H_{40}N_2O_4S_4$ H 525.1949; found 525.1960.

Synthesis of networks

Representative synthesis of C_6E-3T 1:1 using DBU

Trimethylolpropane tris(3-mercaptopropionate) (0.266 g, 1.00 molar equiv., 0.67 mmol) was weighed into a 20 mL scintillation vial. A 1.0 mL stock solution of disulfide monomer (1.0 mL, 1.50 molar equiv., 1.00 mmol) was added to the vial and the mixture was lightly mixed. A 100 mg·mL^{-1} stock solution of DBU (10.2 µL, 0.01 molar equiv., 0.067 mmol) was added in one portion, the mixture was vigorously shaken for 5-10 s and then poured onto a glass slide (L × W = 75 mm × 25 mm) to obtain thin films (c.a. 0.1 mm thickness). The film was left overnight (c.a. 16 h) to ensure solvent removal and then peeled off the substrate for analysis. In order to obtain thicker films (c.a. 1 mm thickness), the reaction was proportionally scaled up 3-fold (i.e. 3.0 mL of disulfide stock solution was used), mixed and left in the 20 mL scintillation vial. The vial was covered with an evaporating dish to ensure slower evaporation of the solvent (for more homogeneous film formation) and left overnight (c.a. 16 h) to dry.

Representative synthesis of C_6E-3T 8:1 using DBU

Trimethylolpropane tris(3-mercaptopropionate) (0.033 g, 1.00 molar equiv., 0.083 mmol) was weighed into a 20 mL scintillation vial. A 1.0 mL stock solution of disulfide monomer (1.0 mL, 12.00 molar equiv., 1.00 mmol) was added to the vial and the mixture was lightly mixed. A 100 mg·mL^{-1} stock solution of DBU (1.26 µL, 0.01 molar equiv., 0.00083 mmol) was added in one portion, the mixture was vigorously shaken for 5-10 s and then poured onto a glass slide (L × W = 75 mm × 25 mm). The film was left overnight (*ca*. 16 h) and then peeled off the substrate for analysis.

C_6E-3T Networks

Ratio 1:1 T_g (DSC) = -37 °C. $T_{d,5\%}$ (TGA) = 254 °C. FTIR 1726 cm^{-1} (C=O ester)

Ratio 2:1 T_g (DSC) = -40 °C. $T_{d,5\%}$ (TGA) = 264 °C. FTIR 1726 cm^{-1} (C=O ester)

Ratio 4:1 T_g (DSC) = -42 °C. $T_{d,5\%}$ (TGA) = 275 °C. FTIR 1726 cm^{-1} (C=O ester)

Ratio 8:1 T_g (DSC) = -56 °C. $T_{d,5\%}$ (TGA) = 274 °C. FTIR 1726 cm^{-1} (C=O ester)

Ratio 16:1 T_g (DSC) = -51 °C. $T_{d,5\%}$ (TGA) = 277 °C. FTIR 1726 cm^{-1} (C=O ester)

$C_{TEG}E$-3T Networks

Ratio 1:1 T_g (DSC) = -39 °C. $T_{d,5\%}$ (TGA) = 234 °C. FTIR 1726 cm^{-1} (C=O ester)

Ratio 4:1 T_g (DSC) = -39 °C. $T_{d,5\%}$ (TGA) = 272 °C. FTIR 1726 cm^{-1} (C=O ester)

Ratio 8:1 T_g (DSC) = -33 °C. $T_{d,5\%}$ (TGA) = 277 °C. FTIR 1726 cm^{-1} (C=O ester)

$C_{TEG}A$-3T Networks

Ratio 1:1 T_g (DSC) = -28 °C. $T_{d,5\%}$ (TGA) = 263 °C. FTIR 1732 cm^{-1} (C=O ester) 1644 cm^{-1} (C=O Amide) 3294 cm^{-1} (N-H)

Ratio 4:1 T_g (DSC) = 4 °C. $T_{d,5\%}$ (TGA) = 265 °C. FTIR 1732 cm^{-1} (C=O ester) 1637 cm^{-1} (C=O Amide) 3297 cm^{-1} (N-H)

Ratio 8:1 T_g (DSC) = -8 °C. $T_{d,5\%}$ (TGA) = 266 °C. FTIR 1729 cm^{-1} (C=O ester) 1640 cm^{-1} (C=O Amide) 3292 cm^{-1} (N-H)

NMR Spectra Collected for Monomers

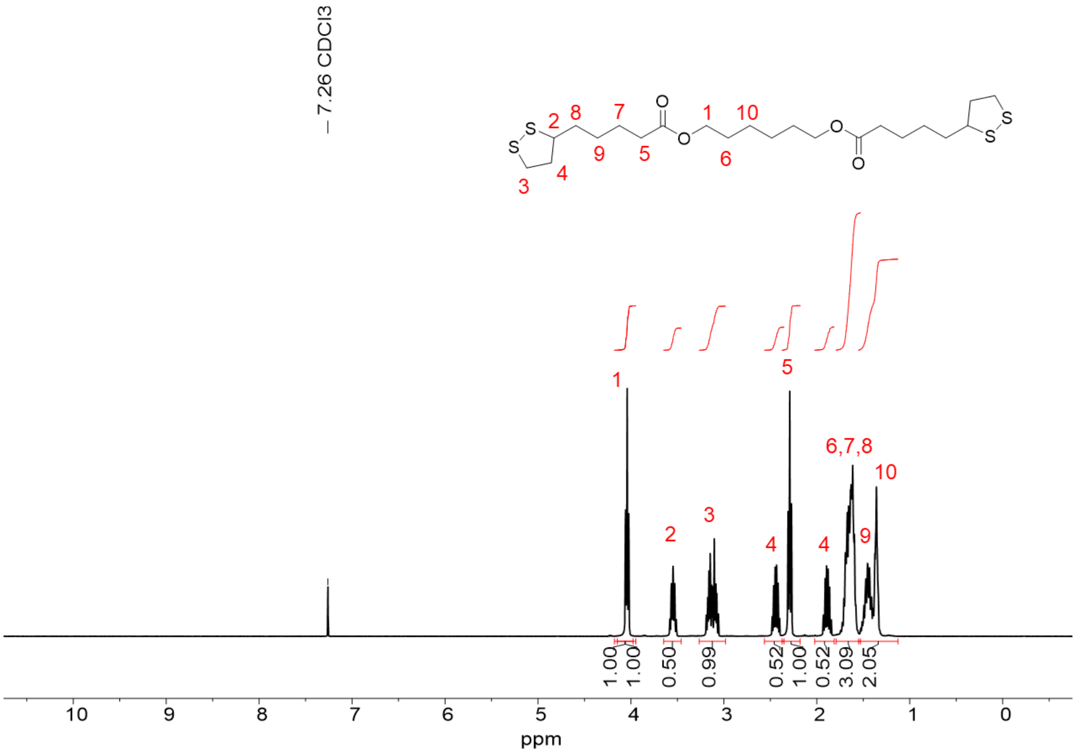

Figure S1. ^{1}H NMR Spectrum of C_6E (400 MHz, 298 K, $CDCl_3$).

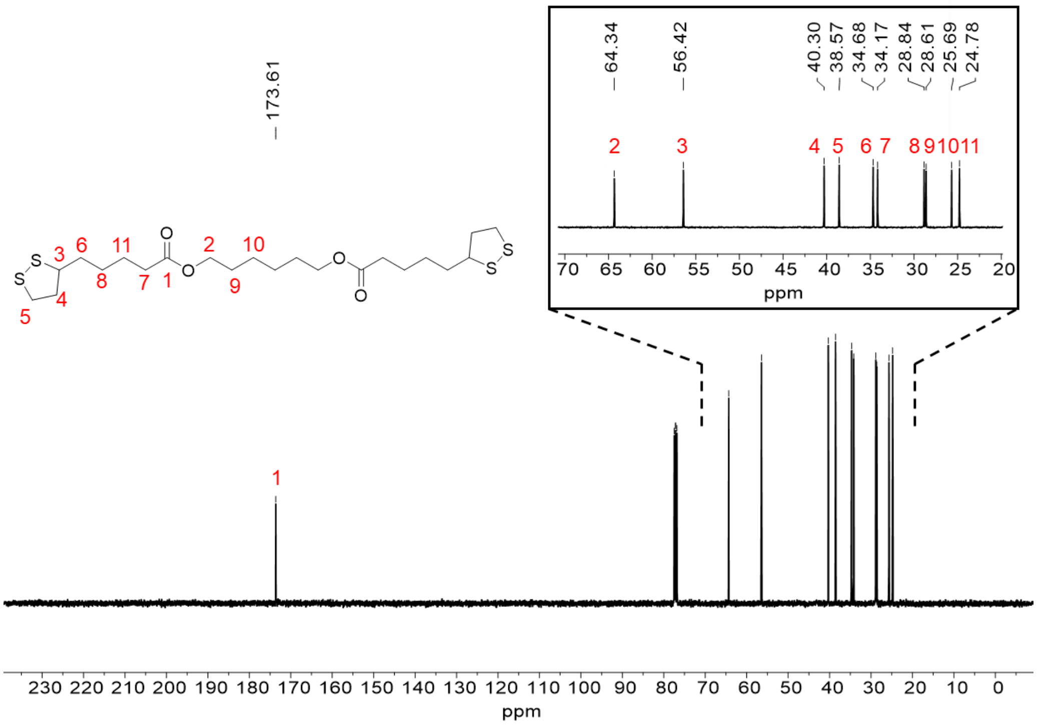

Figure S2. ^{13}C NMR Spectrum of C_6E (101 MHz, 298 K, $CDCl_3$).

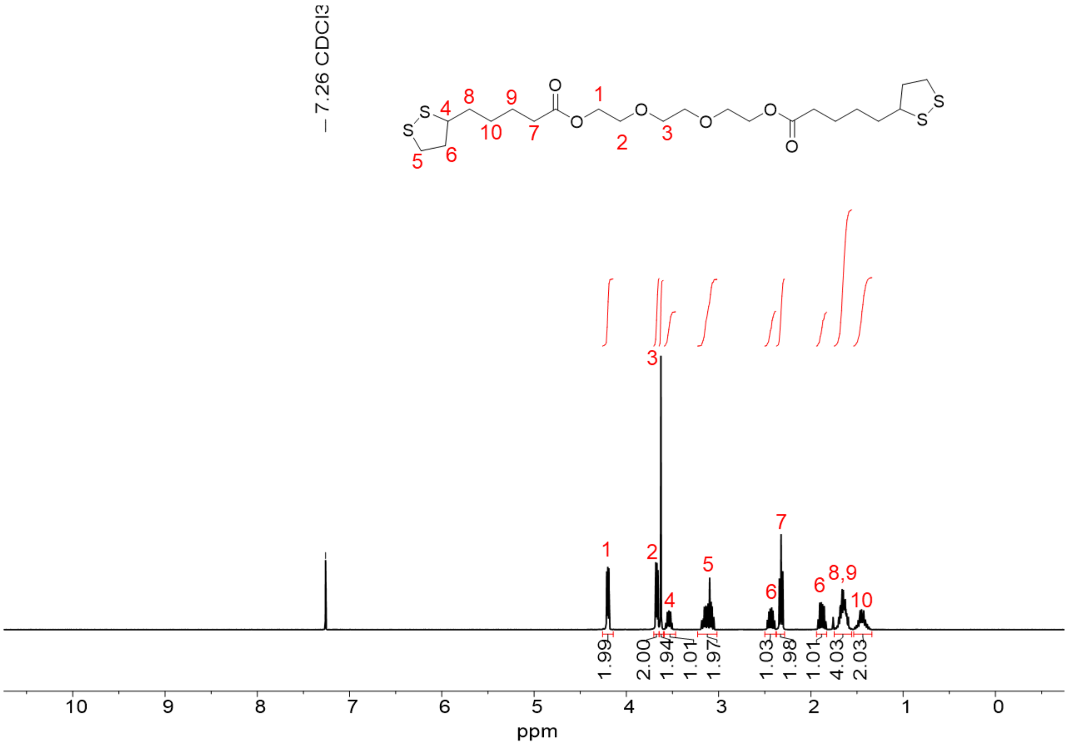

Figure S3. ^{1}H NMR Spectrum of $C_{TEG}E$ (400 MHz, 298 K, $CDCl_3$).

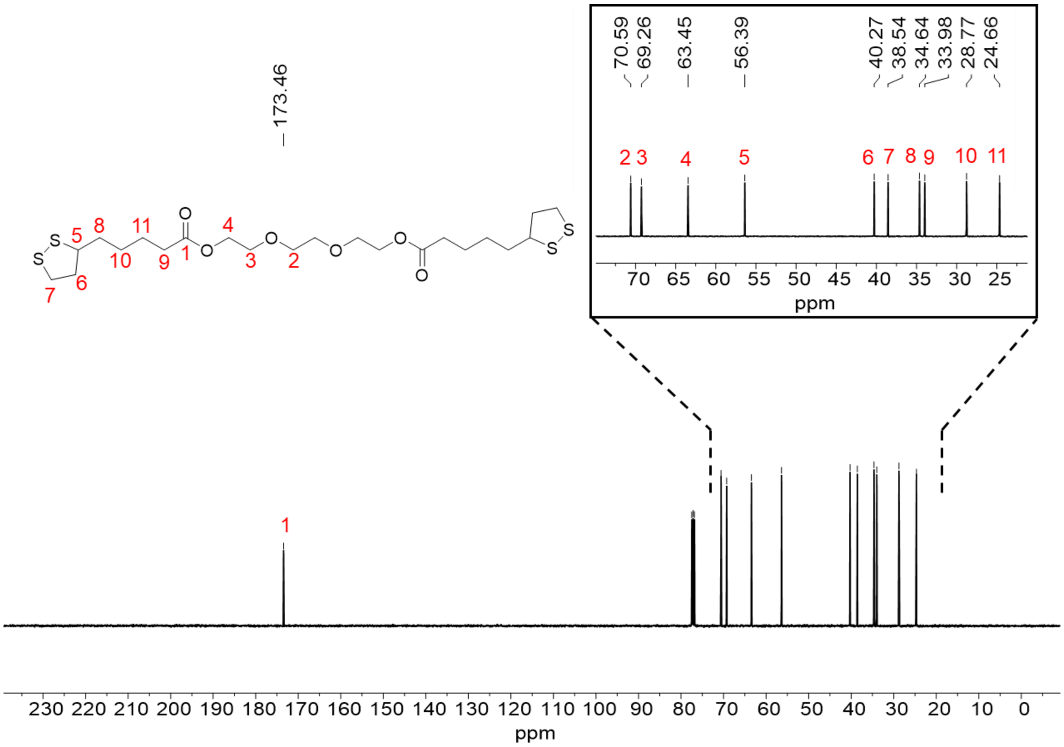

Figure S4. ^{13}C NMR Spectrum of $C_{TEG}E$ (101 MHz, 298 K, $CDCl_3$).

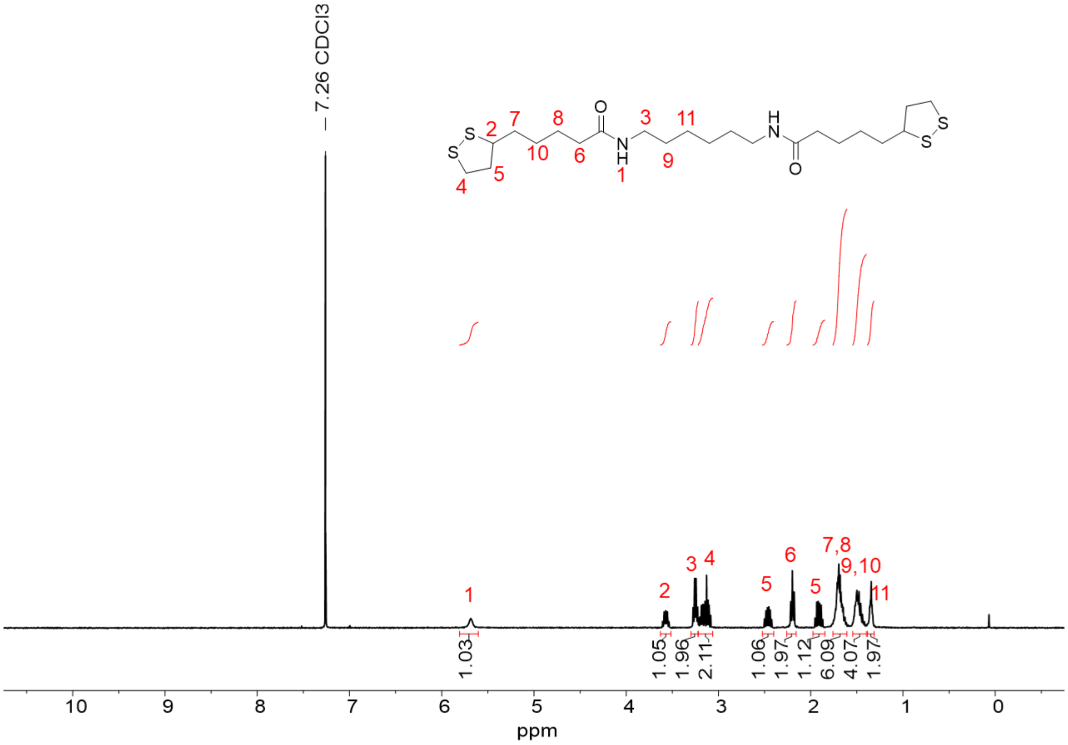

Figure S5. ^{1}H NMR Spectrum of C_6A (400 MHz, 298 K, $CDCl_3$).

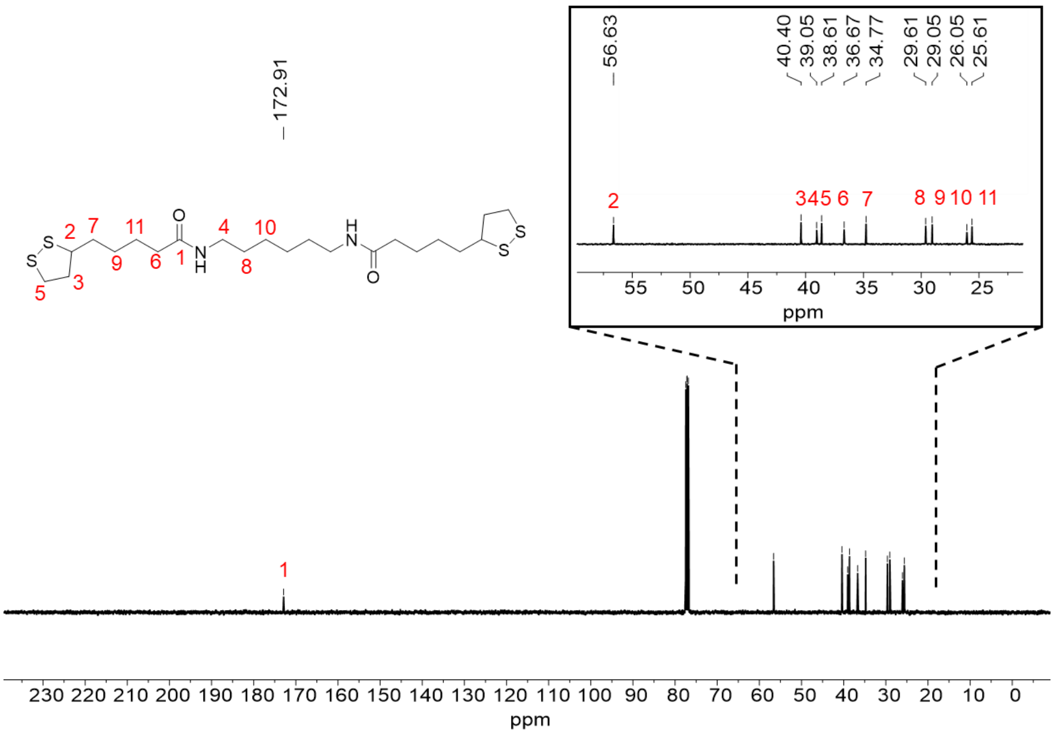

Figure S6. ^{13}C NMR Spectrum of C_6A (101 MHz, 298 K, $CDCl_3$).

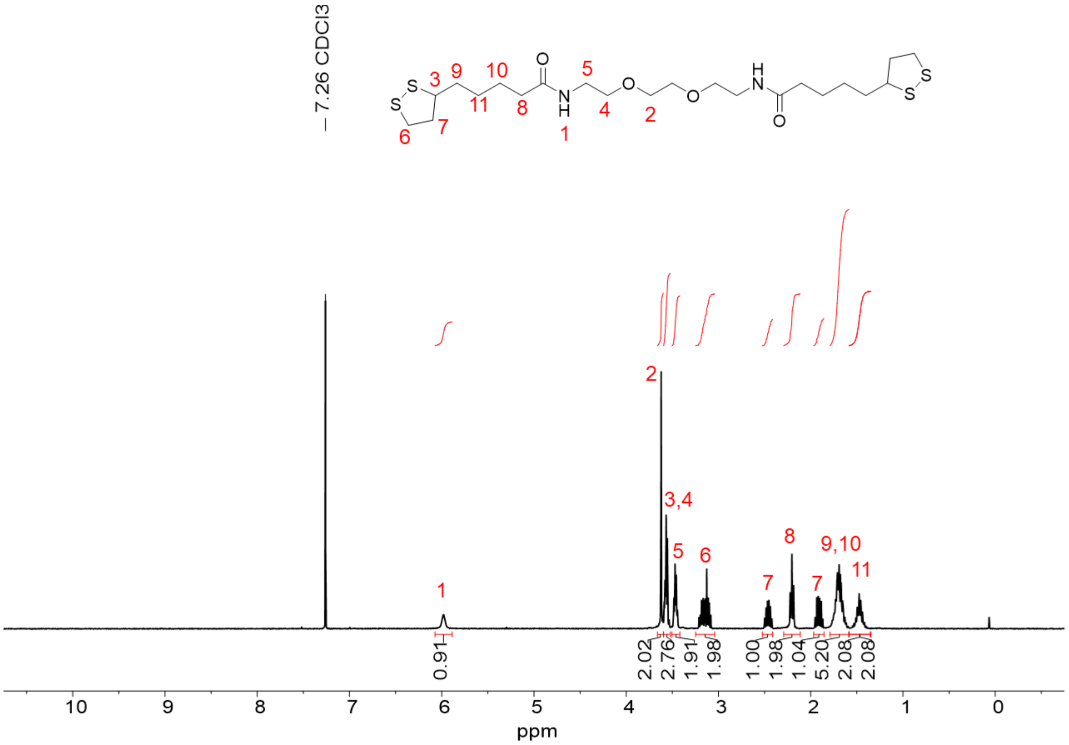

Figure S7. ^{1}H NMR Spectrum of $C_{TEG}A$ (400 MHz, 298 K, $CDCl_3$).

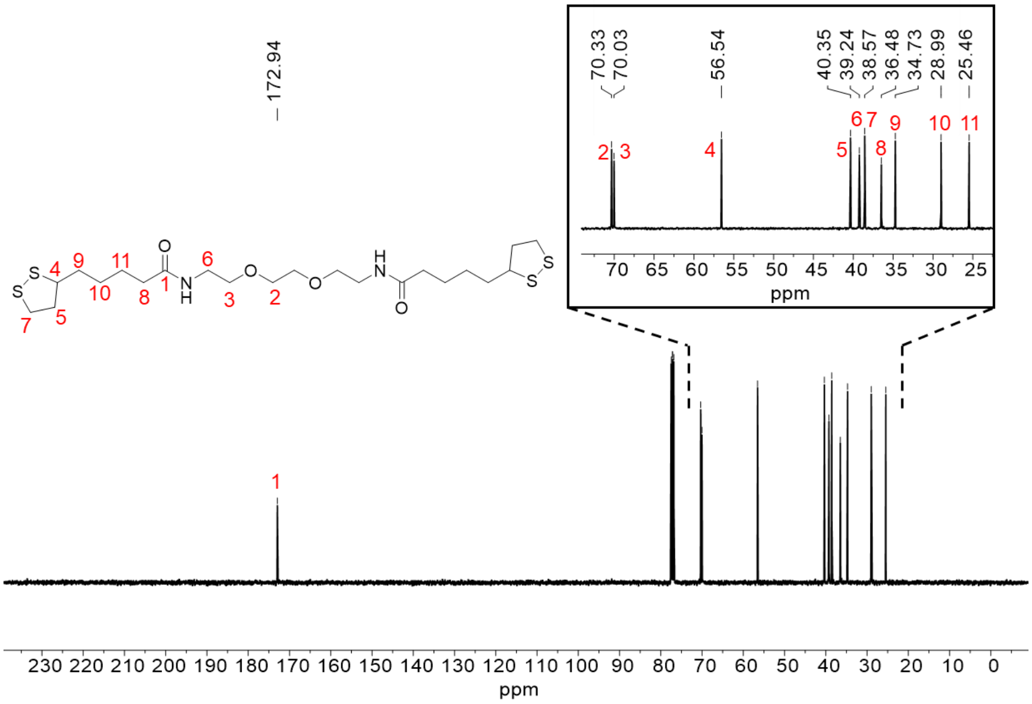

Figure S8. 13C NMR Spectrum of $C_{TEG}A$ (101 MHz, 298 K, $CDCl_3$).

FTIR Data

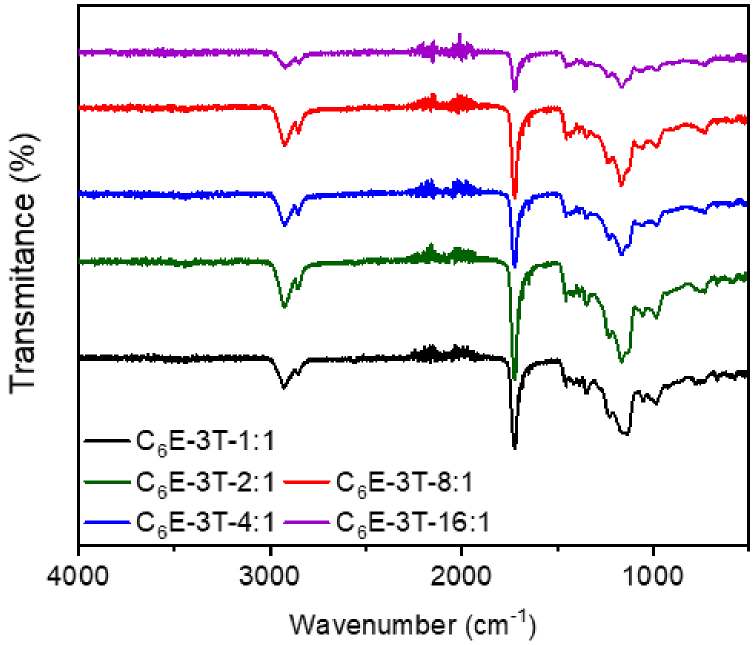

Figure S9. FTIR spectra of C_6E-3T networks.

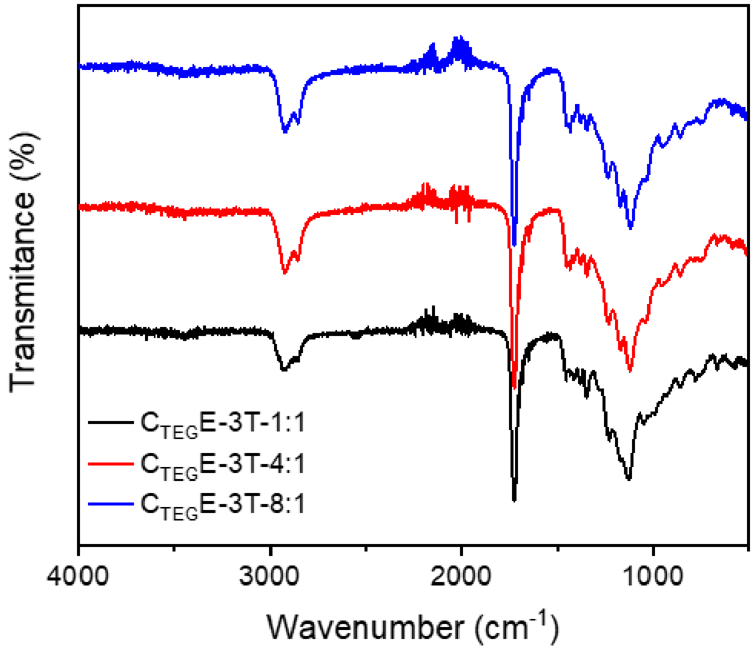

Figure S10. FTIR spectra of $C_{TEG}E$-3T networks.

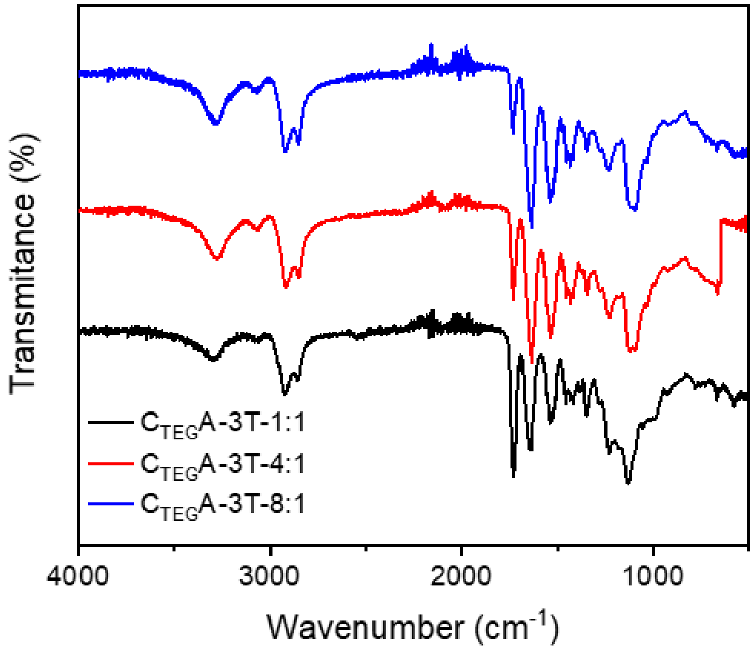

Figure S11. FTIR spectra of $C_{TEG}A$-3T networks.

DSC Data

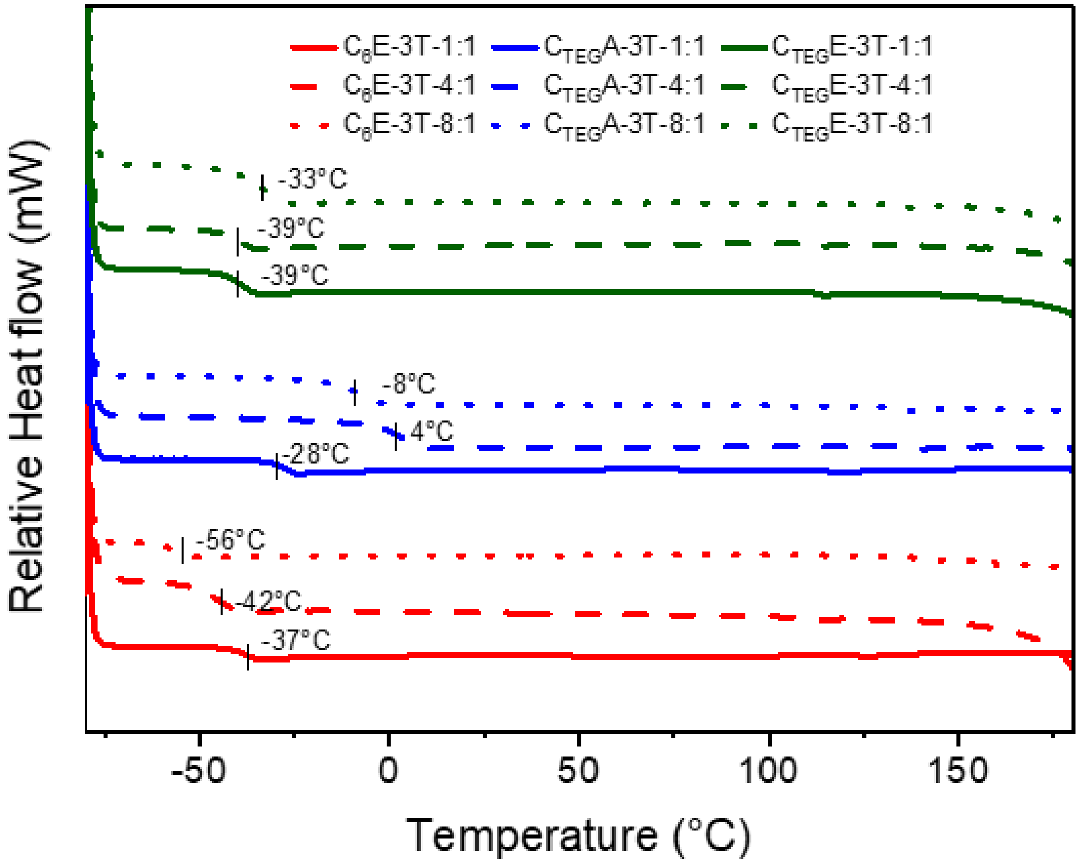

Figure S12. DSC thermograms of 2nd heating cycles for all networks at various ratios from −80 to 180 °C, 10 °C min^{-1}.

TGA Data

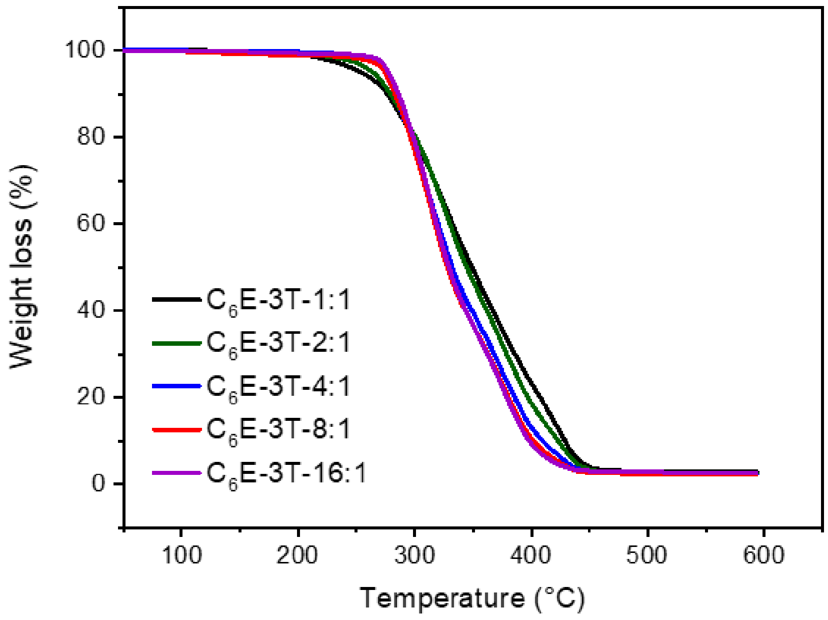

Figure S13. TGA thermograms of C_6E-3T networks.

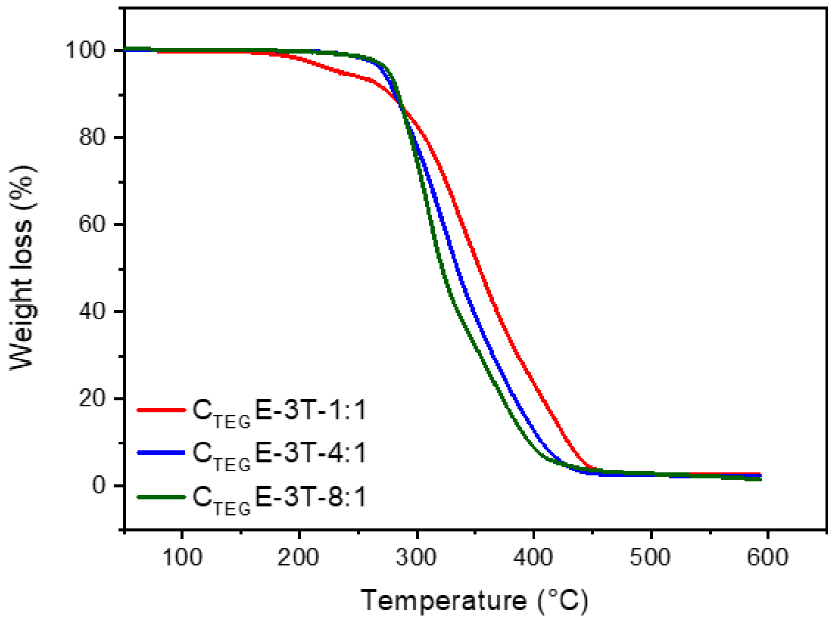

Figure S14. TGA thermograms of $C_{TEG}E$-3T networks.

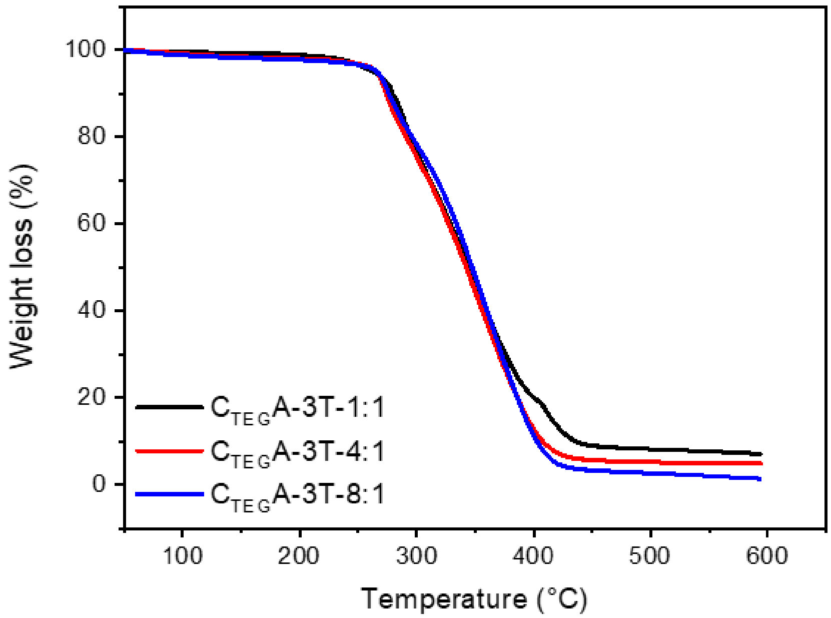

Figure S15. TGA thermograms of C_{TEG}A-3T networks.

Rheology Data

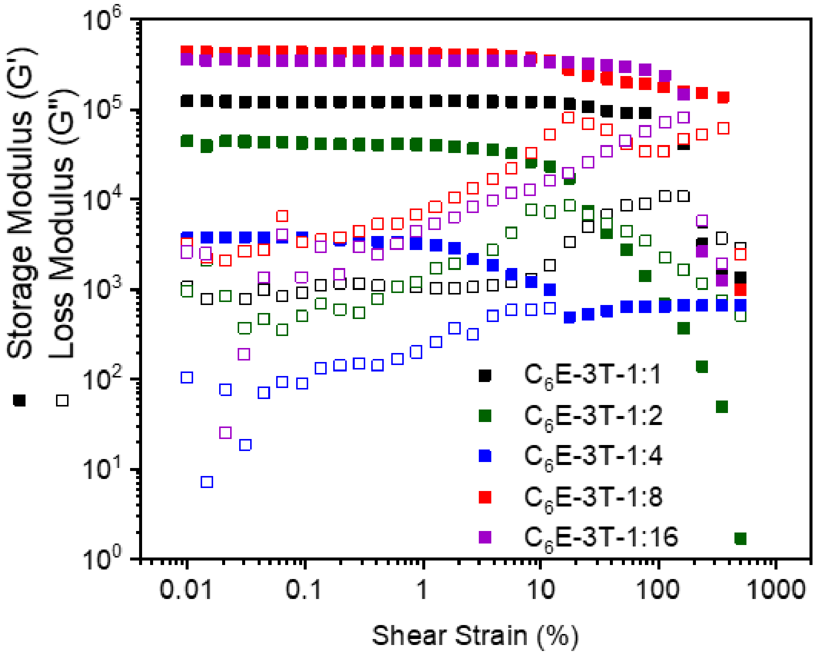

Figure S16. Strain sweeps of C_6E-3T networks.

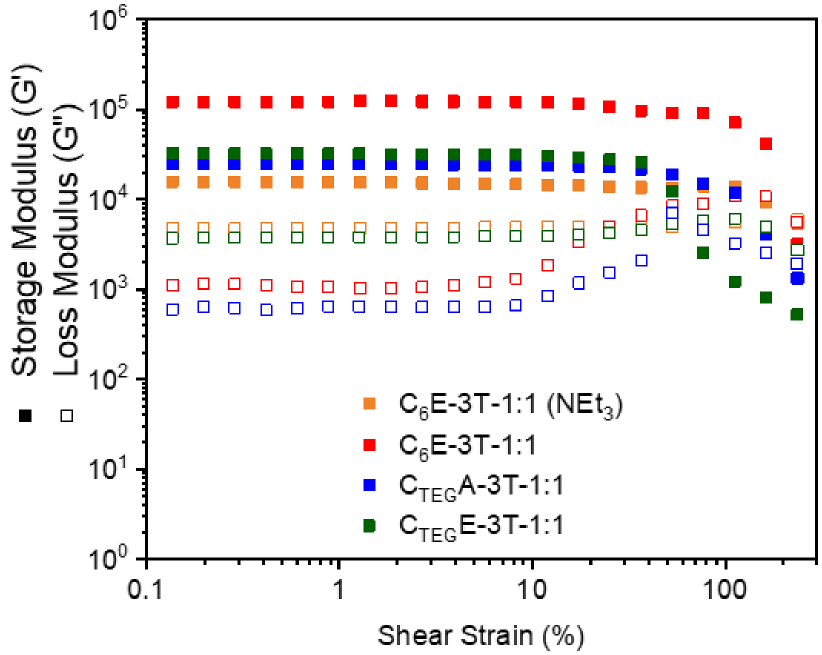

Figure S17. Strain sweeps of all networks at 1:1 ratio.

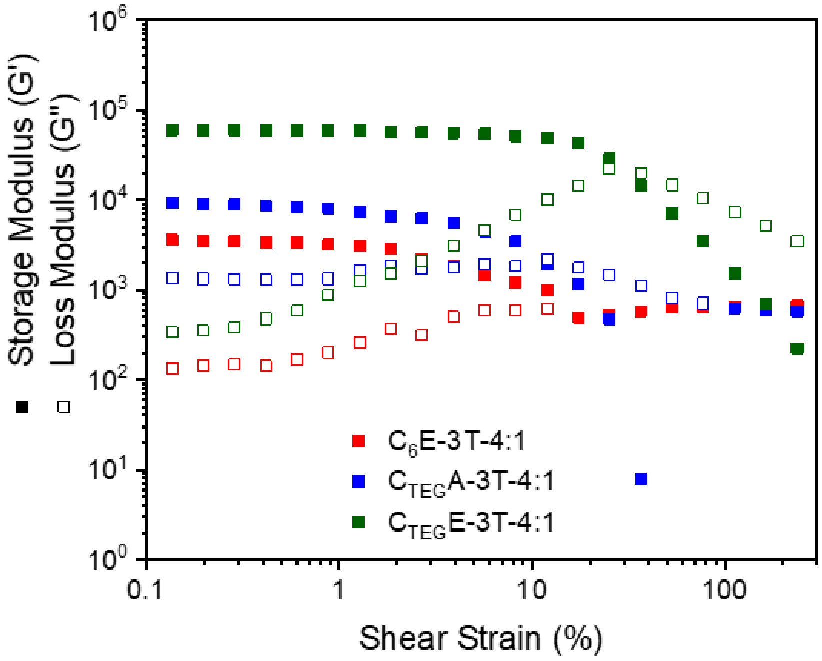

Figure S18. Strain sweeps of all networks at 4:1 ratio.

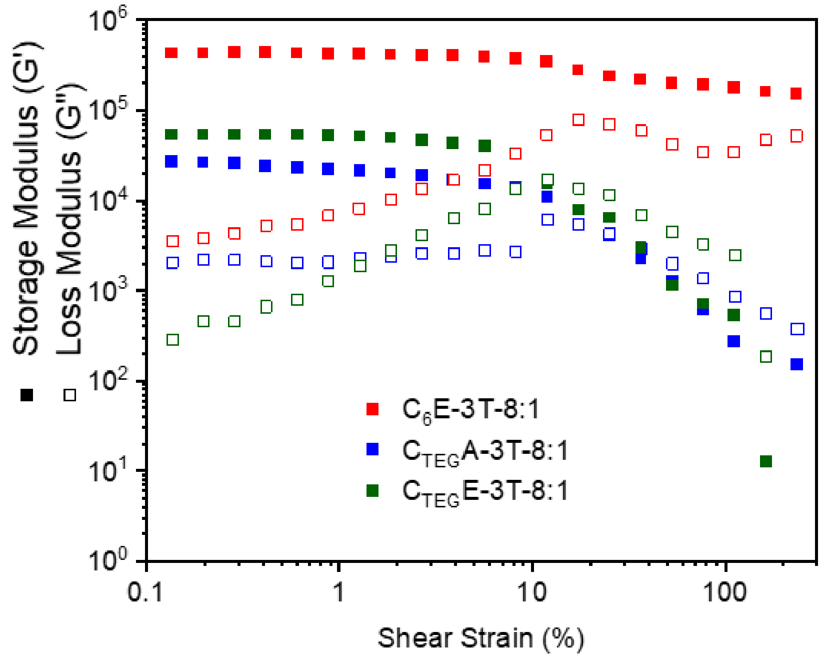

Figure S19. Strain sweeps of all networks at 8:1 ratio.

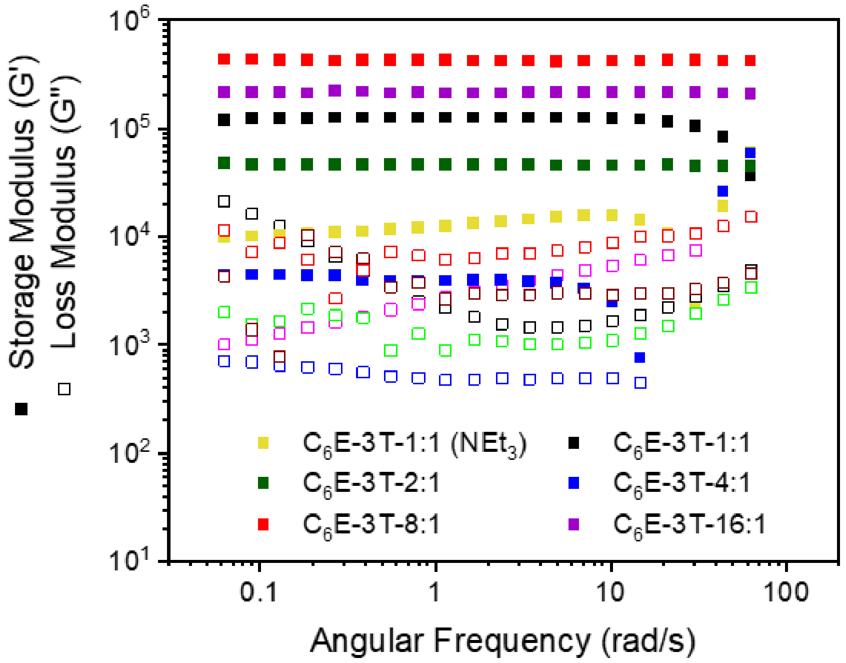

Figure S20. Frequency sweeps of all C_6E-3T networks.

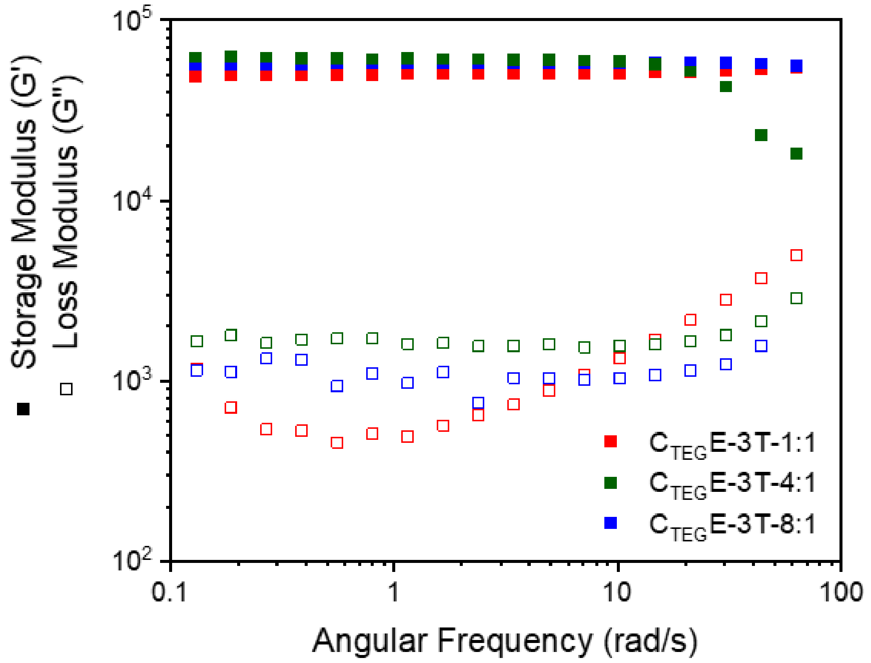

Figure S21. Frequency sweeps of C_{TEG}E-3T networks.

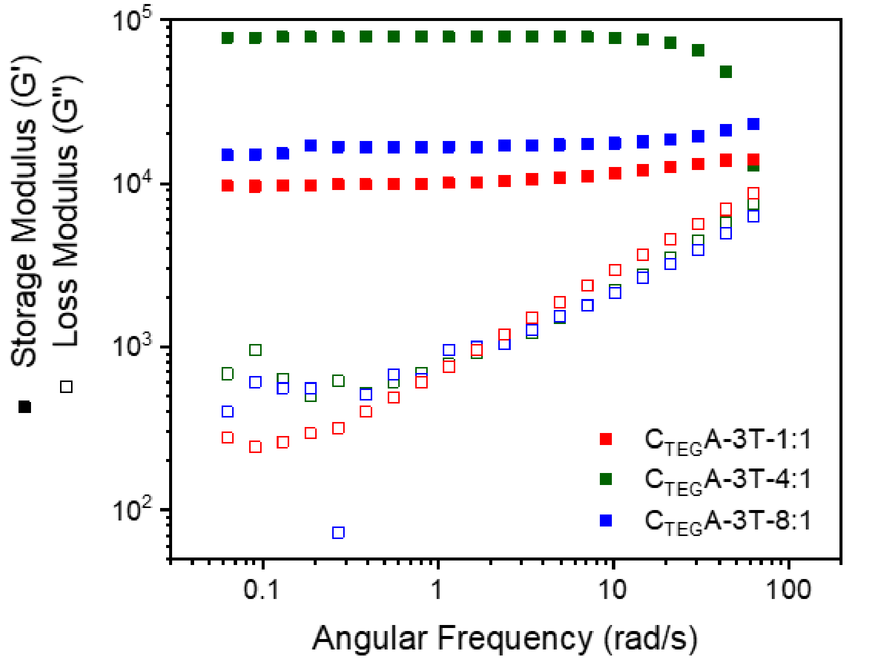

Figure S22. Frequency sweeps of C_{TEG}A-3T networks.

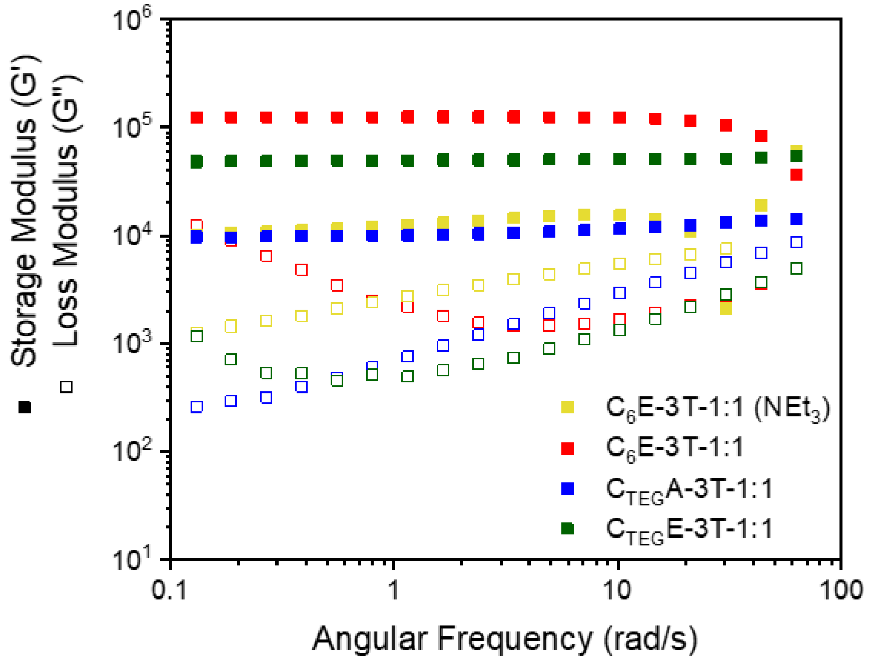

Figure S23. Frequency sweeps of all networks at 1:1 ratio.

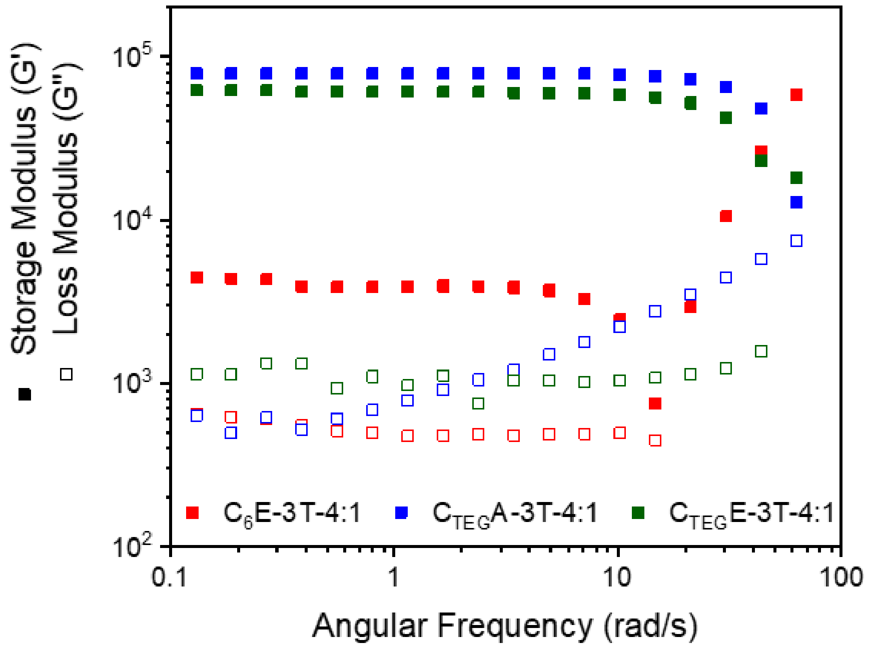

Figure S24. Frequency sweeps of all networks at 4:1 ratio.

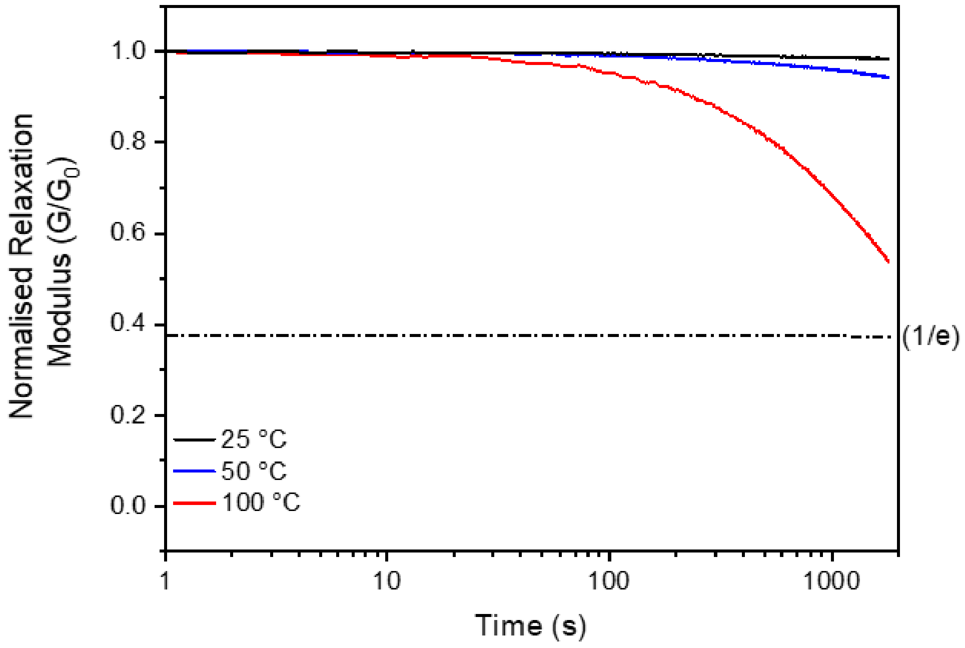

Figure S25. Stress relaxation of the CTEGE-3T-8:1 network at various temperatures.

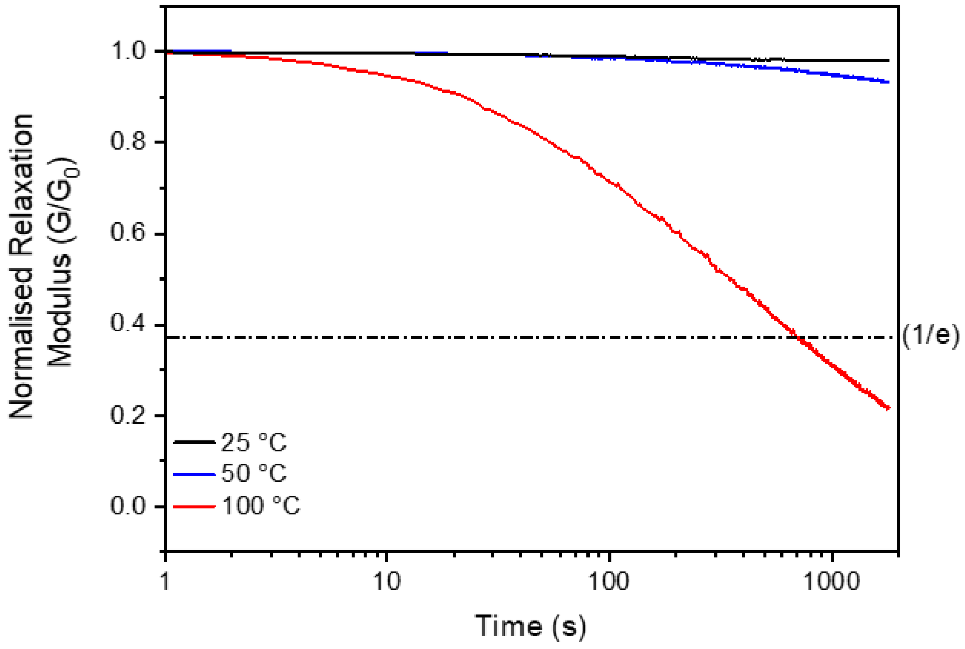

Figure S26. Stress relaxation of the CTEGA-3T-8:1 network at various temperatures.

Thermal reprocessing of films

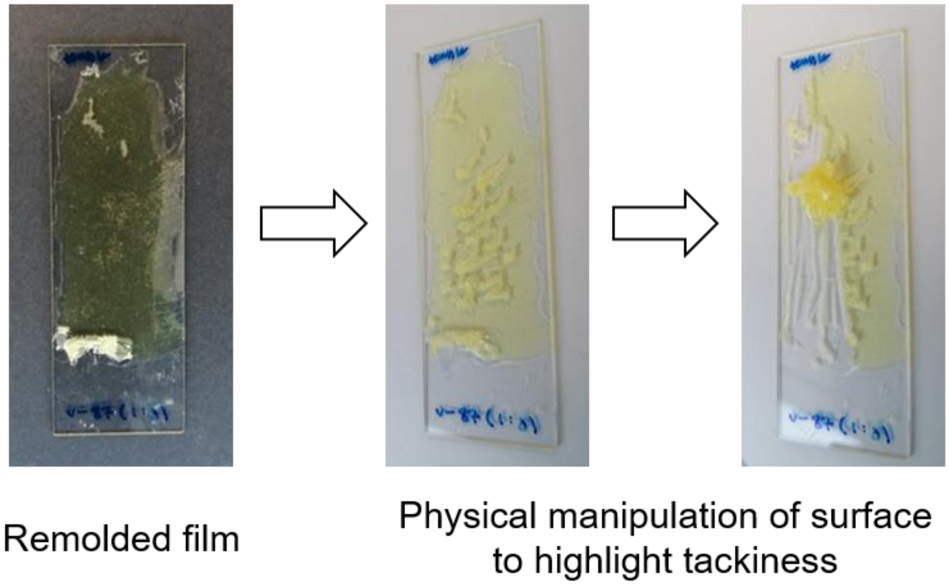

Figure S27. Photographs of C_6A-3T-8:1 network that was thermally reprocessed at 180 °C.

Chemical recycling of films

Table S1. Summary of chemical recycling experiments for C_6E-3T networks at 22 °C (40 mg/mL).

Network ratio (disulfide:thiol)	Mass (mg)	[a]Volume of solution (mL)	Concentration of solution (M)	[b]Approximate time for full dissolution (s)
1:1	200	5	0.01	210
1:1	150	3.75	0.05	95
8:1	200	5	0.01	180
8:1	150	3.75	0.05	90
8:1 (reprocessed film)	150	3.75	0.05	130

[a]Volume was adjusted for experiments to ensure same overall concentration of network relative to solvent. [b]Estimated time at which no network particles were visible.

DMA Data

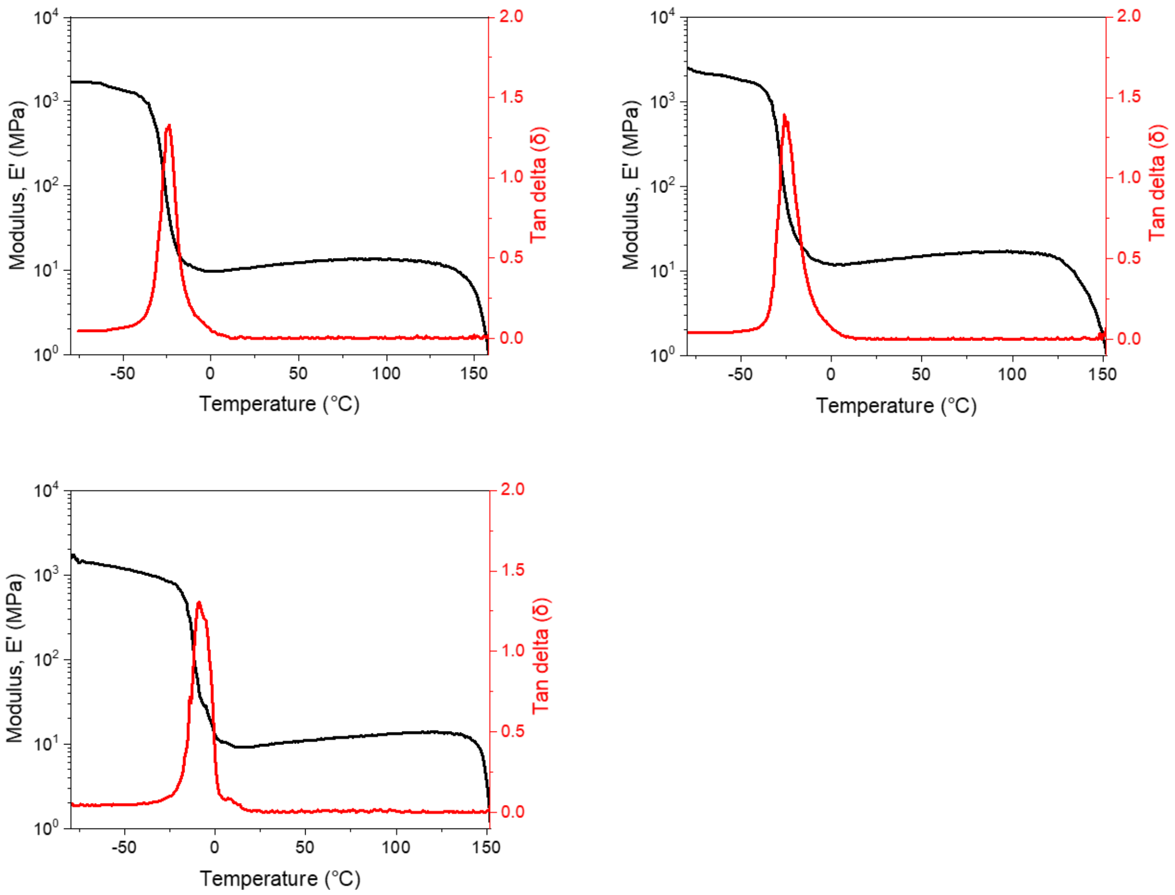

Figure S28. DMA temperature sweep for C_6E-3T-8:1 (n = 3). Summary of results are shown in Table S3.

Summary of Network Properties

Table S2. Physical, thermal and mechanical properties for networks with varied compositions.

Network	Ratio	^aT_g (DSC)	^bT_d (5% loss)	$^c\tau$ at 50 °C (s)	$^c\tau$ at 100 °C (s)	^{d}Storage Modulus (kPa)
C_6E-3T	1:1	−37	254			120
	2:1	−40	264			47
	4:1	−42	275			4.4
	8:1	−56	274	**	80	431
	16:1	−51	277			213
C_{TEG}E-3T	1:1	−39	234			48
	4:1	−39	272			62
	8:1	−33	277	**	**	56
C_{TEG}A-3T	1:1	−28	263			9.6
	4:1	4	265			78
	8:1	−8	266	**	708	15

[a]Glass transition temperature determined from 2nd DSC heating run. [b]Thermal degradation temperature at 5% weight loss determined from TGA [c]Relaxation time determined to be the time required to reach 37% (1/e) of the initial stress value on normalized relaxation modulus (G/G_0) based on Maxwell model[1] (**relaxation time was not reached within 1800s). [d]Storage modulus determined from the dynamic strain sweep on rheometer.

Table S3. Summary of DMA and tensile data for C_6E-3T-8:1 network.

Network	Ratio	^aT_g (DMA)	^{b}Storage Modulus, *E'*, (MPa) at −70 °C	^{b}Storage Modulus, *E'*, (MPa) at 25 °C	^{c}Flow Temp. (°C)	^{d}Youngs Modulus (MPa)	^{d}Strain at break (%)	^{d}Stress at break (MPa)
C_6E-3T	8:1	−20 ± 8	1748 ± 294	11.2 ± 1.2	145 ± 8	9.1 ± 1.9	19 ± 2.2	1.8 ± 0.4

[a]Glass transition temperature determined from tan δ of DMA temperature sweep from −80 to 180 °C, 5 °C min^{-1}. [b]Storage modulus determined during DMA temperature sweep at −70 °C and 25 °C. [c]Flow temperature determined from DMA temperature sweep and calculated by the 1st derivative of the modulus versus temperature after the rubbery plateau region. [d]Young's modulus, strain at break and stress at break determined from uniaxial tensile testing at 22 °C, 10 mm min^{-1} strain rate. All uncertainties are reported as 1 S.D.

References

1. B. M. El-Zaatari, J. S. A. Ishibashi and J. A. Kalow, *Polym. Chem.*, 2020, **11**, 5339-5345.
